# MAVE-NN: learning genotype-phenotype maps from multiplex assays of variant effect

**DOI:** 10.1186/s13059-022-02661-7

**Published:** 2022-04-15

**Authors:** Ammar Tareen, Mahdi Kooshkbaghi, Anna Posfai, William T. Ireland, David M. McCandlish, Justin B. Kinney

**Affiliations:** 1 Simons Center for Quantitative Biology, Cold Spring Harbor Laboratory, Cold Spring Harbor, NY 11724 USA; 2 Present Address: Regeneron Pharmaceuticals, Inc., Tarrytown, NY 10591 USA; 3 Department of Physics, California Institute of Technology, Pasadena, CA 91125 USA; 4 Present Address: Department of Applied Physics, Harvard University, Cambridge, MA 02134 USA

## Abstract

Multiplex assays of variant effect (MAVEs) are a family of methods that includes deep mutational scanning experiments on proteins and massively parallel reporter assays on gene regulatory sequences. Despite their increasing popularity, a general strategy for inferring quantitative models of genotype-phenotype maps from MAVE data is lacking. Here we introduce MAVE-NN, a neural-network-based Python package that implements a broadly applicable information-theoretic framework for learning genotype-phenotype maps—including biophysically interpretable models—from MAVE datasets. We demonstrate MAVE-NN in multiple biological contexts, and highlight the ability of our approach to deconvolve mutational effects from otherwise confounding experimental nonlinearities and noise.

**Supplementary Information:**

The online version contains supplementary material available at 10.1186/s13059-022-02661-7.

## Background

**Fig. 1:**
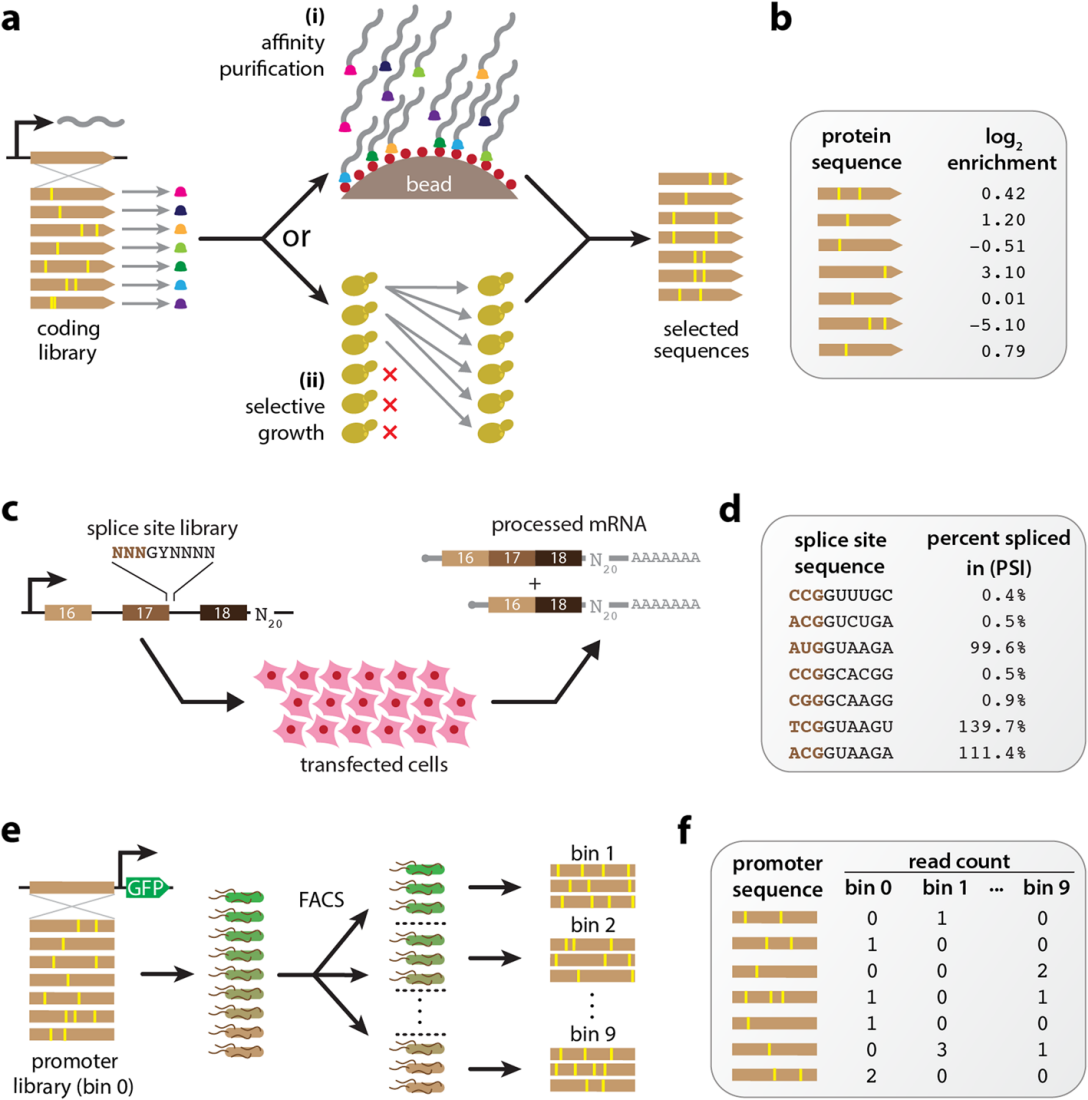
Diverse MAVEs and the datasets they produce. **a** DMS assays using either affinity purification or selective growth. **(i)** The DMS assay of Olson et al. [8] used a library of variant GB1 proteins physically linked to their coding mRNAs. Functional GB1 proteins were then enriched using IgG beads. **(ii)** The DMS studies of Seuma et al. [9] and Bolognesi et al. [10] used selective growth in genetically modified *Saccharomyces cerevisiae* to assay the functionality of variant Aβ and TDP-43 proteins, respectively. In all three experiments, deep sequencing was used to determine an enrichment ratio for each protein variant. **b** The resulting DMS datasets consist of variant protein sequences and their corresponding log enrichment values. **c** The MPSA of Wong et al. [11]. A library of 3-exon minigenes was constructed from exons 16, 17, and 18 of the human *BRCA2* gene, with each minigene having a variant 5′ss at exon 17 and a random 20 nt barcode in the 3′ UTR. This library was transfected into HeLa cells, and deep sequencing of RNA barcodes was used to quantify mRNA isoform abundance. **d** The resulting MPSA dataset comprises variant 5′ss with (noisy) PSI values. **e** The sort-seq MPRA of Kinney et al. [12]. A plasmid library was generated in which randomly mutagenized versions of the *Escherichia coli lac* promoter drove the expression of GFP. Cells carrying these plasmids were sorted using FACS, and the variant promoters in each bin of sorted cells, as well as the initial library, were sequenced. **f** The resulting dataset comprises a list of variant promoter sequences, as well as a matrix of counts for each variant in each FACS bin. MAVE: multiplex assay of variant effect; DMS: deep mutational scanning; GB1: protein G domain B1; IgG: immunoglobulin G; Aβ: amyloid beta; TDP-43: TAR DNA-binding protein 43; MPSA: massively parallel splicing assay; *BRCA2*: breast cancer 2; 5′ss: 5′ splice site(s); UTR: untranslated region; PSI: percent spliced in; GFP: green fluorescent protein; FACS: fluorescence-activated cell sorting

Over the last decade, the ability to quantitatively study genotype-phenotype (G-P) maps has been revolutionized by the development of multiplex assays of variant effect (MAVEs), which can measure molecular phenotypes for thousands to millions of genotypic variants in parallel [1, 2]. MAVE is an umbrella term that describes a diverse set of experimental methods, some examples of which are illustrated in Fig. 1. Deep mutational scanning (DMS) experiments [3] are a type of MAVE commonly used to study protein sequence-function relationships. These assays work by linking variant proteins to their coding sequences, either directly or indirectly, then using deep sequencing to assay which variants survive a process of activity-dependent selection (e.g., Fig. 1a). Massively parallel reporter assays (MPRAs) are another major class of MAVE and are commonly used to study DNA or RNA sequences that regulate gene expression at a variety of steps, including transcription, mRNA splicing, cleavage and polyadenylation, translation, and mRNA decay [4–7]. MPRAs typically rely on either an RNA-seq readout of barcode abundances (Fig. 1c) or the sorting of cells expressing a fluorescent reporter gene (Fig. 1e).

Most computational methods for analyzing MAVE data have focused on accurately quantifying the activity of individual assayed sequences [13–19]. However, MAVE measurements like enrichment ratios or cellular fluorescence levels usually cannot be interpreted as providing direct quantification of biologically meaningful activities, due to the presence of experiment-specific nonlinearities and noise. Moreover, MAVE data is usually incomplete, as one often wishes to understand G-P maps over vastly larger regions of sequence space than can be exhaustively assayed. The explicit quantitative modeling of G-P maps can address both the indirectness and incompleteness of MAVE measurements [1, 20]. The goal here is to determine a mathematical function that, given a sequence as input, will return a quantitative value for that sequence’s molecular phenotype. Such quantitative modeling has been of great interest since the earliest MAVE methods were developed [12, 21, 22], but no general-use software has yet been described for inferring G-P maps of arbitrary functional form from the diverse types of datasets produced by commonly used MAVEs.

Here we introduce a unified conceptual framework for the quantitative modeling of MAVE data. This framework is based on the use of latent phenotype models, which assume that each assayed sequence has a well-defined latent phenotype (specified by the G-P map), of which the MAVE experiment provides a noisy indirect readout (described by the measurement process). The quantitative forms of both the G-P map and the measurement process are then inferred from MAVE data simultaneously. We further introduce an information-theoretic approach for separately assessing the performance of the G-P map and the measurement process components of latent phenotype models. This strategy is implemented in an easy-to-use open-source Python package called MAVE-NN, which represents latent phenotype models as neural networks and infers the parameters of these models from MAVE data using a TensorFlow 2 backend [23].

In what follows, we expand on this unified MAVE modeling strategy and apply it to a diverse array of DMS and MPRA datasets. Doing so, we find that MAVE-NN provides substantial advantages over other MAVE modeling approaches. In particular, we illustrate how the ability of MAVE-NN to train custom G-P maps can shed light on the biophysical mechanisms of protein function and gene regulation. We also highlight the substantial benefits of including sequence variants with multiple mutations in assayed sequence libraries, as doing so allows MAVE-NN to deconvolve the features of the G-P map from potentially confounding effects of experimental nonlinearities and noise. Indeed, including just a modest number of multiple-mutation variants in a MAVE experiment can be beneficial even when one is primarily interested in the effects of single mutations.

## Results

### Latent phenotype modeling strategy

**Fig. 2:**
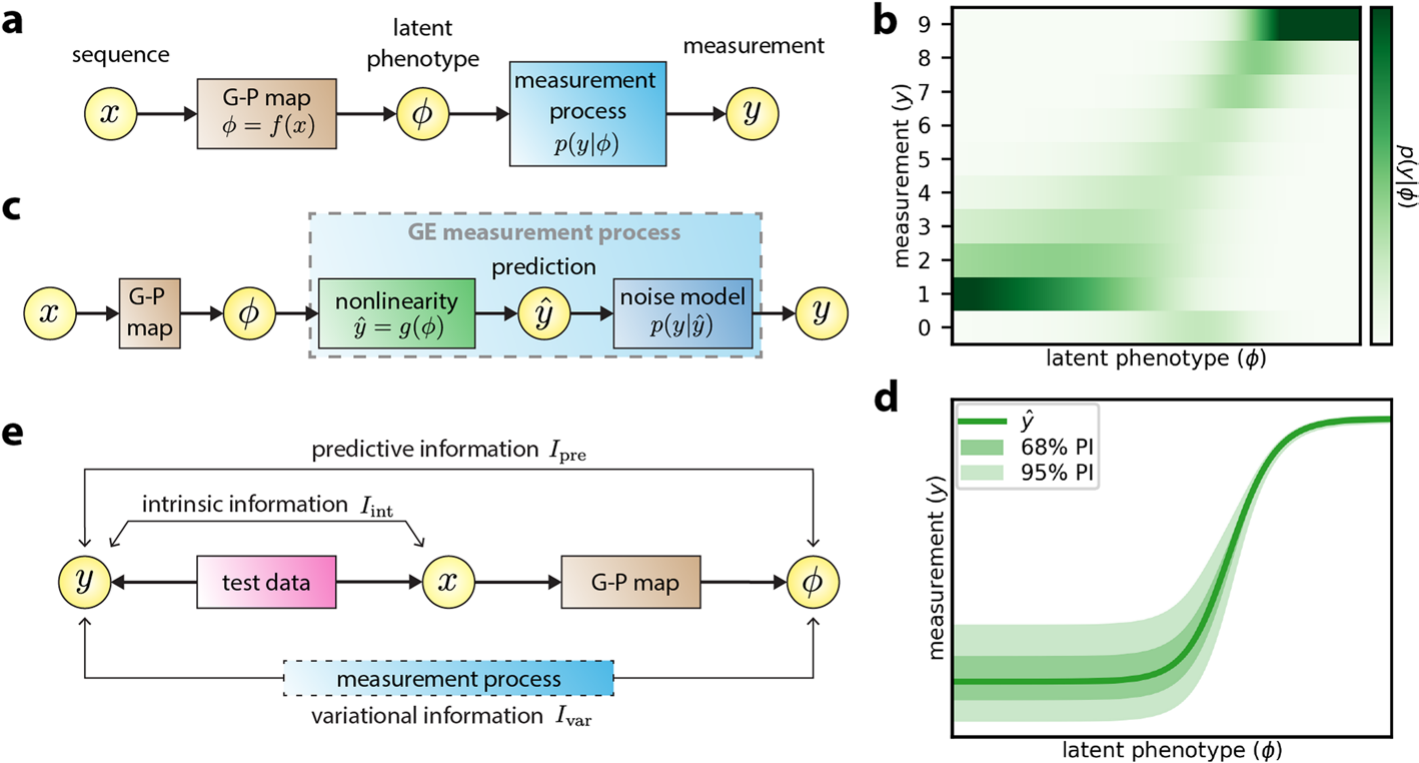
MAVE-NN quantitative modeling strategy. **a** Structure of latent phenotype models. A deterministic G-P map *f*(*x*) maps each sequence *x* to a latent phenotype *ϕ*, after which a probabilistic measurement process *p*(*y*| *ϕ*) generates a corresponding measurement *y*. **b** Example of an MPA measurement process inferred from the sort-seq MPRA data of Kinney et al. [12]. MPA measurement processes are used when *y* values are discrete. **c** Structure of a GE regression model, which is used when *y* is continuous. A GE measurement process assumes that the mode of *p*(*y*| *ϕ*), called the prediction $\hat{y}$, is given by a nonlinear function *g*(*ϕ*), and the scatter about this mode is described by a noise model $p\left(y|\hat{y}\right)$. **d** Example of a GE measurement process inferred from the DMS data of Olson et al. [8]. Shown are the nonlinearity, the 68% PI, and the 95% PI. **e** Information-theoretic quantities used to assess model performance. Intrinsic information, *I*_int_, is the mutual information between sequences *x* and measurements *y*. Predictive information, *I*_pre_, is the mutual information between measurements *y* and the latent phenotype values *ϕ* assigned by a G-P map. Variational information, *I*_var_, is a linear transformation of the log likelihood of a full latent phenotype model. The model performance inequality, *I*_int_ ≥ *I*_pre_ ≥ *I*_var_, always holds on test data (modulo finite data uncertainties), with *I*_int_ = *I*_pre_ when the G-P map is correct, and *I*_pre_ = *I*_var_ when the measurement process correctly describes the distribution of *y* conditioned on *ϕ*. G-P: genotype-phenotype; MPA: measurement process agnostic; MPRA: massively parallel reporter assay; GE: global epistasis; DMS: deep mutational scanning; PI: prediction interval

MAVE-NN supports the analysis of MAVE data on DNA, RNA, and protein sequences and can accommodate either continuous or discrete measurement values. Given a set of sequence-measurement pairs, MAVE-NN aims to infer a probabilistic mapping from sequences to measurements. Our primary enabling assumption, which is encoded in the structure of the latent phenotype model (Fig. 2a), is that this mapping occurs in two stages. Each sequence is first mapped to a latent phenotype by a deterministic G-P map. This latent phenotype is then mapped to possible measurement values via a stochastic measurement process. During training, the G-P map and measurement process are simultaneously learned by maximizing a regularized form of likelihood.

MAVE-NN includes four types of built-in G-P maps: additive, neighbor, pairwise, and black box. Additive G-P maps assume that each character at each position within a sequence contributes independently to the latent phenotype. Neighbor G-P maps incorporate interactions between adjacent (i.e., nearest neighbor) characters in a sequence, while pairwise G-P maps include interactions between all pairs of characters in a sequence regardless of the distance separating the characters in each pair. Black box G-P maps have the form of a densely connected multilayer perceptron, the specific architecture of which can be controlled by the user. MAVE-NN also supports custom G-P maps that can be used, e.g., to represent specific biophysical hypotheses about the mechanisms of sequence function.

To handle both discrete and continuous measurement values, two different strategies for modeling measurement processes are provided. Measurement process agnostic (MPA) regression uses techniques from the biophysics literature [12, 20, 24, 25] to analyze MAVE datasets that report discrete measurements. Here the measurement process is represented by an overparameterized neural network that takes the latent phenotype as input and outputs the probability of each possible categorical measurement (Fig. 2b). Global epistasis (GE) regression (Fig. 2c), by contrast, leverages ideas previously developed in the evolution literature for analyzing datasets that contain continuous measurements [26–29], and is becoming an increasingly popular strategy for modeling DMS data [30–33]. Here, the latent phenotype is nonlinearly mapped to a prediction that represents the most probable measurement value. A noise model is then used to describe the distribution of likely deviations from this prediction. MAVE-NN supports both homoscedastic and heteroscedastic noise models based on three different classes of probability distribution: Gaussian, Cauchy, and skewed-*t*. In particular, the skewed-*t* distribution, introduced by Jones and Faddy [34], reduces to Gaussian and Cauchy distributions in certain limits, but also accommodates asymmetric experimental noise. Figure 2d shows an example of a GE measurement process with a heteroscedastic skewed-*t* noise model.

Readers should note that the current implementation of MAVE-NN places constraints on input data and model architecture. Input sequences must be the same length, and when analyzing continuous data, only scalar measurements (as opposed to vectors of multiple measurements) can be used to train models. In addition, because our method for learning the form of experimental nonlinearities depends on observing how multiple mutations combine, MAVE-NN’s functionality is more limited when analyzing MAVE libraries that contain only single-mutation variants. More information on these constraints and the reasons behind them can be found below in the section “Constraints on datasets and models”.

### Information-theoretic measures of model performance

We further propose three distinct quantities for assessing the performance of latent phenotype models: intrinsic information, predictive information, and variational information (Fig. 2e). These quantities come from information theory and are motivated by thinking of G-P maps in terms of information compression. In information theory, a quantity called mutual information quantifies the amount of information that the value of one variable communicates about the value of another [35, 36]. Mutual information is symmetric, is nonnegative, and is measured in units called “bits.” If the two variables in question are independent, their mutual information will be zero bits. If instead, knowing the value of one of these variables allows you to narrow down the value of the other variable to one of two otherwise equally likely possibilities, their mutual information will be 1.0 bits. This mutual information will be 2.0 bits if this second variable’s value is narrowed down to one of four possibilities, 3.0 bits if it is narrowed down to one of eight possibilities, and so on. But importantly, mutual information does not require that the relationship between the two variables of interest be so clean cut, and can in fact be computed between any two types of variables—discrete, continuous, multi-dimensional, etc.. This property makes the information-based quantities we propose applicable to all MAVE datasets, regardless of the specific type of experimental readout used. By contrast, many of the standard model performance metrics have restricted domains of applicability: accuracy can only be applied to data with categorical labels, Pearson and Spearman correlation can only be applied to data with univariate continuous labels, and so on. We note, however, that estimating mutual information and related quantities from finite data is nontrivial and that MAVE-NN uses a variety of approaches to do this.

Our information metrics are as follows. Intrinsic information, *I*_int_, is the mutual information between the sequences and measurements contained within a MAVE dataset. This quantity provides a useful benchmark against which to compare the performance of inferred G-P maps. Predictive information, *I*_pre_, is the mutual information between MAVE measurements and the latent phenotype values predicted by an inferred G-P map. This quantifies how well the latent phenotype preserves sequence-encoded information that is determinative of experimental measurements. When evaluated on test data, *I*_pre_ is bounded above by *I*_int_, and equality is realized only when the G-P map losslessly compresses sequence-encoded information. Variational information, *I*_var_, is a linear transformation of log likelihood (or equivalently, cross-entropy) that provides a variational lower bound on *I*_pre_ [37–39]. The difference between *I*_pre_ and *I*_var_ quantifies how accurately the inferred measurement process matches the observed distribution of measurements conditioned on latent phenotypes (see Additional file 1: Section S1).

MAVE-NN infers model parameters by maximizing an (often quite lightly) regularized form of likelihood. These computations are performed using the standard backpropagation-based training algorithms provided within TensorFlow 2. With certain caveats noted (see “Methods”), this optimization procedure maximizes *I*_pre_ while avoiding the costly estimates of mutual information at each iteration that have hindered the adoption of previous mutual-information-based modeling strategies [12, 25].

### Application: deep mutational scanning assays

We now demonstrate the capabilities of MAVE-NN on three DMS datasets, starting with the study of Olson et al. [8]. These authors measured the effects of single and double mutations to residues 2–56 of the IgG binding domain of protein G (GB1). To assay the binding of GB1 variants to IgG, the authors combined mRNA display with ultra-high-throughput DNA sequencing [Fig. 1a(i)]. The resulting dataset reports log enrichment values for all possible 1045 single mutations and 530,737 (nearly all possible) double mutations to this 55 aa protein sequence (Fig. 1b).

**Fig. 3:**
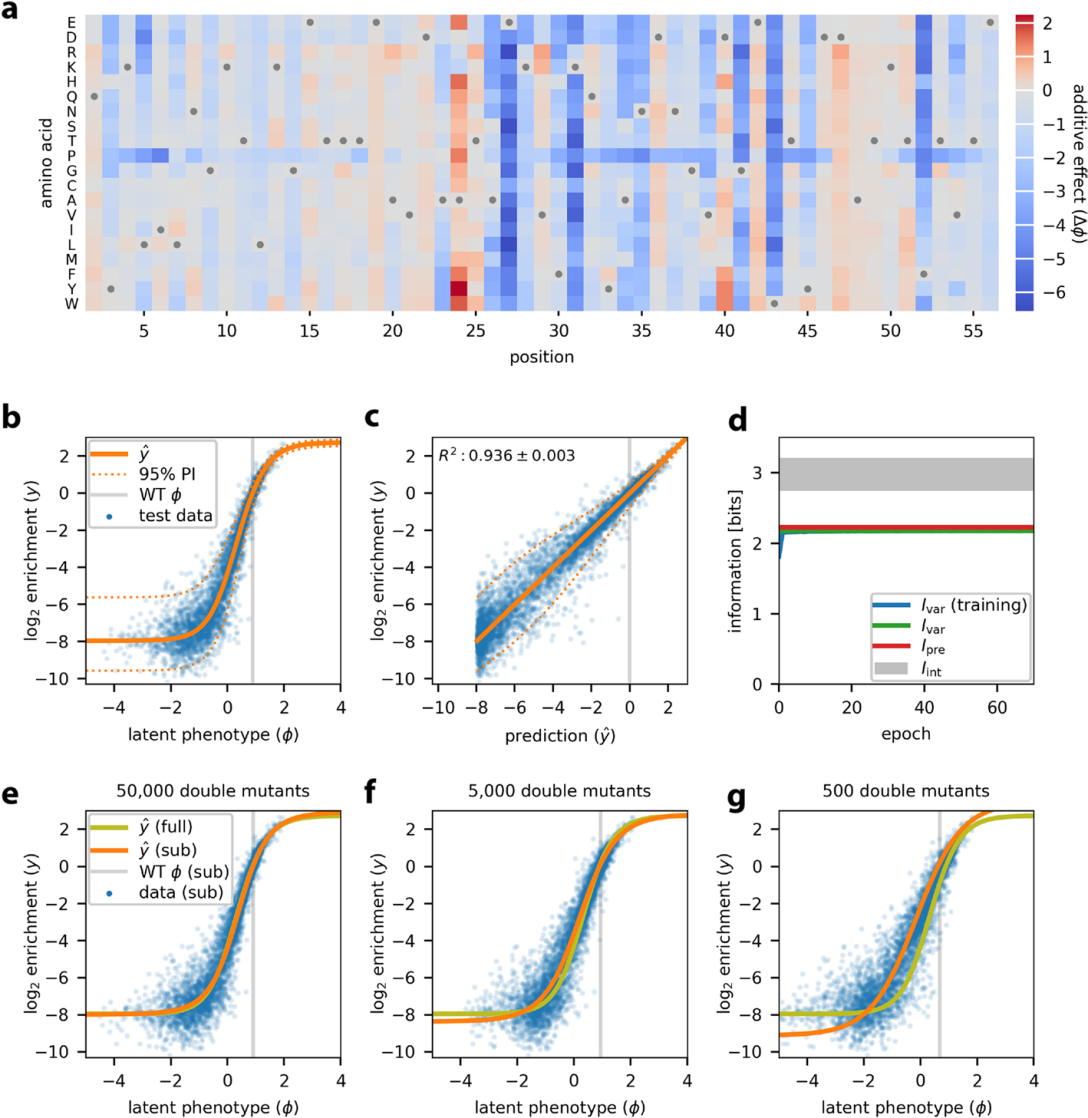
Analysis of DMS data for protein GB1. MAVE-NN was used to infer a latent phenotype model, consisting of an additive G-P map and a GE measurement process with a heteroscedastic skewed-*t* noise model, from the DMS data of Olson et al. [8]. All 530,737 pairwise variants reported for positions 2 to 56 of the GB1 domain were analyzed. Data were split 90:5:5 into training, validation, and test sets. **a** The inferred additive G-P map parameters. Gray dots indicate wildtype residues. Amino acids are ordered as in Olson et al. [8]. **b** GE plot showing measurements versus predicted latent phenotype values for 5000 randomly selected test set sequences (blue dots), alongside the inferred nonlinearity (solid orange line) and the 95% PI (dotted orange lines) of the noise model. Gray line indicates the latent phenotype value of the wildtype sequence. **c** Measurements plotted against $\hat{y}$ predictions for these same sequences. Dotted lines indicate the 95% PI of the noise model. Gray line indicates the wildtype value of $\hat{y}$. Uncertainty in the value of *R*^2^ reflects standard error. **d** Corresponding information metrics computed during model training (using training data) or for the final model (using test data). The uncertainties in these estimates are very small—roughly the width of the plotted lines. Gray shaded area indicates allowed values for intrinsic information based on the upper and lower bounds estimated as described in “Methods.” **e–g** Test set predictions (blue dots) and GE nonlinearities (orange lines) for models trained using subsets of the GB1 data containing all single mutants and 50,000 (**e**), 5000 (**f**), or 500 (**g**) double mutants. The GE nonlinearity from panel **b** is shown for reference (yellow-green lines). DMS: deep mutational scanning; GB1: protein G domain B1; GE: global epistasis; G-P: genotype-phenotype; PI: prediction interval

Inspired by the work of Otwinowski et al. [29], we used MAVE-NN to infer a latent phenotype model comprising an additive G-P map and a GE measurement process. This inference procedure required only about 5 min on one node of a computer cluster (Additional file 1: Fig. S1e). Figure 3a illustrates the inferred additive G-P map via the effects that every possible single-residue mutation has on the latent phenotype. From this heatmap of additive effects, we can immediately identify all of the critical GB1 residues, including the IgG-interacting residues at 27, 31, and 43 [8]. We also observe that missense mutations to proline throughout the GB1 domain tend to negatively impact IgG binding, as expected due to this amino acid’s exceptional conformational rigidity.

Figure 3b illustrates the corresponding GE measurement process, revealing a sigmoidal relationship between log enrichment measurements and the latent phenotype values predicted by the G-P map. Nonlinearities like this are ubiquitous in DMS data due to the presence of background and saturation effects. Unless they are explicitly accounted for in one’s quantitative modeling efforts, as they are here, these nonlinearities can greatly distort the parameters of inferred G-P maps. Figure 3c shows that accounting for this nonlinearity yields predictions that correlate quite well with measurement values.

One particularly useful feature of MAVE-NN is that every inferred model can be used as a MAVE dataset simulator (see “Methods”). Among other things, this capability allows users to verify whether MAVE-NN can recover ground truth models from realistic datasets in diverse biological contexts. By analyzing simulated data generated by the model we inferred for GB1, we observed that MAVE-NN could indeed accurately and robustly recover both the GE nonlinearity and the ground truth G-P map parameters (Additional file 1: Fig. S1a-d).

Figure 3d summarizes the values of our information-theoretic metrics for model performance. On held-out test data, we find that *I*_var_ = 2.178 ± 0.027 bits and *I*_pre_ = 2.225 ± 0.017 bits, where the uncertainties in these values reflect standard errors. The similarity of these two values suggests that the inferred GE measurement process, which includes a heteroscedastic skewed-*t* noise model, very well describes the distribution of residuals. We further find that 2.741 ± 0.013 bits ≤ *I*_int_ ≤ 3.215 ± 0.007 bits (see “Methods”), meaning that the inferred G-P map accounts for 69–81% of the total sequence-dependent information in the dataset. While this performance is impressive, the additive G-P map evidently misses some relevant aspect of the true genetic architecture. This observation motivates the more complex biophysical model for GB1 discussed later in “Results.”

The ability of MAVE-NN to deconvolve experimental nonlinearities from additive G-P maps requires that some of the assayed sequences contain multiple mutations. This is because such nonlinearities are inferred by reconciling the effects of single mutations with the effects observed for combinations of two or more mutations. To investigate how many multiple-mutation variants are required, we performed GE inference on subsets of the GB1 dataset containing all 1045 single-mutation sequences and either 50,000, 5000, or 500 double-mutation sequences (see “Methods”). The shapes of the resulting GE nonlinearities are illustrated in Fig. 3e–g. Remarkably, MAVE-NN is able to recover the underlying nonlinearity using only about 500 randomly selected double mutants, which represent only ~0.1% of all possible double mutants. The analysis of simulated data also supports the ability to accurately recover ground truth model predictions using highly reduced datasets (Additional file 1: Fig. S1f). These findings have important implications for the design of DMS experiments: even if one only wants to determine an additive G-P map, including a modest number of multiple-mutation sequences in the assayed library is often advisable because it an enable the removal of artifactual nonlinearities.

To test the capabilities of MAVE-NN on less complete DMS datasets, we analyzed recent experiments on amyloid beta (Aβ) [9] and TDP-43 [10], both of which exhibit aggregation behavior in the context of neurodegenerative diseases. In these experiments, protein functionality was assayed using selective growth (Fig. 1a(ii)) in genetically modified *Saccaromyces cerevisiae*: Seuma et al. [9] positively selected for Aβ aggregation, whereas Bolognesi et al. [10] selected against TDP-43 toxicity. Like with GB1, the variant libraries used in these two experiments included a substantial number of multiple-mutation sequences: 499 single- and 15,567 double-mutation sequences for Aβ; 1266 single- and 56,730 double-mutation sequences for TDP-43. But unlike with GB1, these datasets are highly incomplete due to the use of mutagenic PCR (for Aβ) or doped oligo synthesis (for TDP-43) in the construction of variant libraries.

**Fig. 4:**
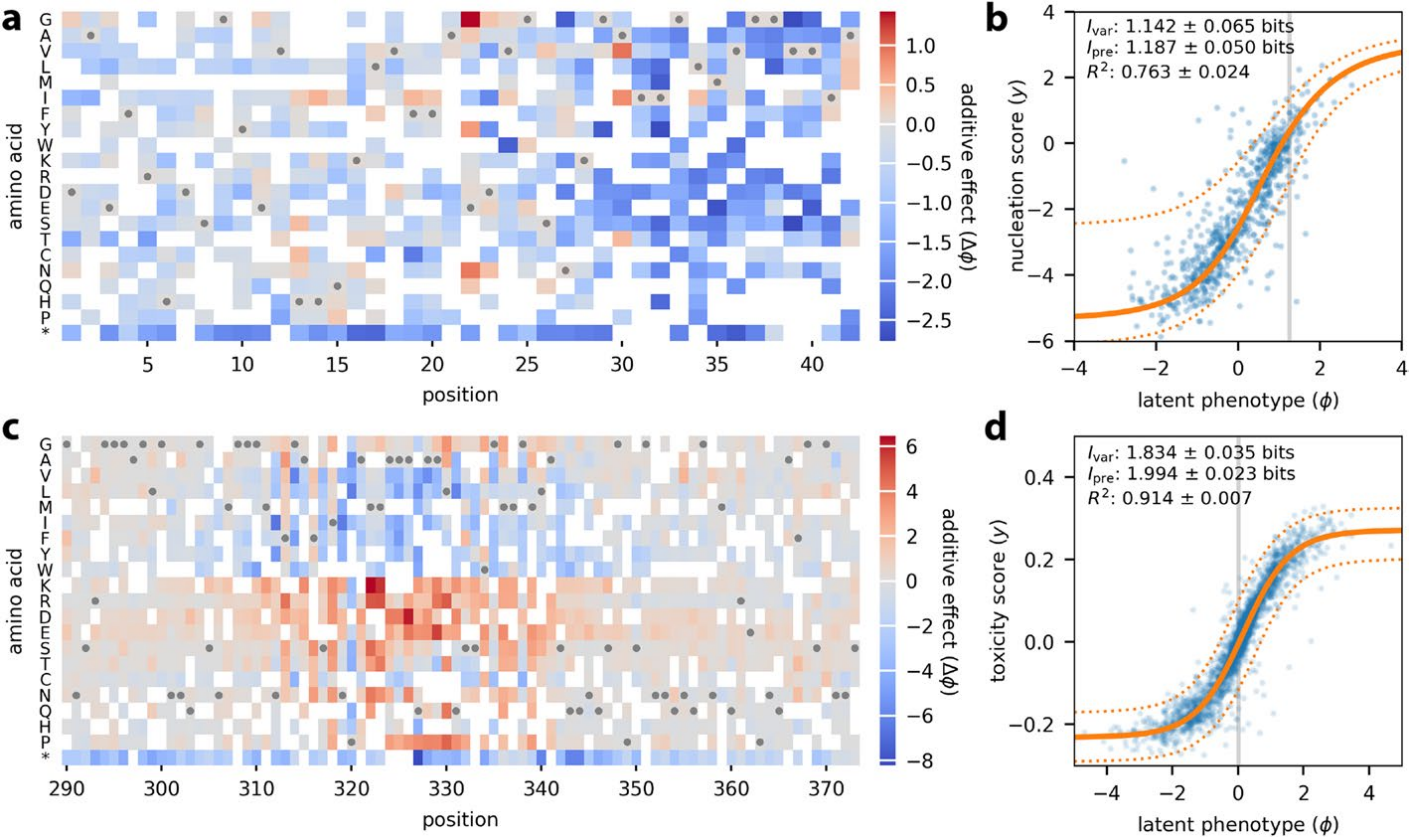
Analysis of DMS data for Aβ and TDP-43. **a, b** Seuma et al. [9] measured nucleation scores for 499 single mutants and 15,567 double mutants of Aβ. These data were used to train a latent phenotype model comprising **a** an additive G-P map and **b** a GE measurement process with a heteroscedastic skewed-*t* noise model. **c, d** Bolognesi et al. [10] measured toxicity scores for 1266 single mutants and 56,730 double mutants of TDP-43. The resulting data were used to train **c** an additive G-P map and **d** a GE measurement process of the same form as in panel **b**. In both cases, data were split 90:5:5 into training, validation, and test sets. In **a, c**, gray dots indicate the wildtype sequence and * indicates a stop codon. White squares [355/882 (40.2%) for Aβ; 433/1764 (24.5%) for TDP-43] indicate residues that were not observed in the training set and thus could not be assigned values for their additive effects. Amino acids are ordered as in the original publications [9, 10]. In **b, d**, blue dots indicate latent phenotype values plotted against measurements for held-out test data. Gray line indicates the latent phenotype value of the wildtype sequence. Solid orange line indicates the GE nonlinearity, and dotted orange lines indicate a corresponding 95% PI for the inferred noise model. Values for *I*_var_, *I*_pre_, and *R*^2^ (between *y* and $\hat{y}$) are also shown. Uncertainties reflect standard errors. Additional file 1: Fig. S2 shows measurements plotted against the $\hat{y}$ predictions of these models. DMS: deep mutational scanning; Aβ: amyloid beta; TDP-43: TAR DNA-binding protein 43; G-P: genotype-phenotype; GE: global epistasis; PI: prediction interval

We used MAVE-NN to infer additive G-P maps from these two datasets, adopting the same type of latent phenotype model used for GB1. Figure 4a illustrates the additive G-P map inferred from aggregation measurements of Aβ variants. In agreement with the original study, we see that most amino acid mutations between positions 30–40 have a negative effect on variant enrichment, suggesting that this region plays a major role in promoting nucleation. Figure 4b shows the corresponding measurement process (see also Additional file 1: Fig. S2). Even though these data are much sparser than the GB1 data, the inferred model performs well on held-out test data (*I*_var_ = 1.142 ± 0.065 bits, *I*_pre_ = 1.187 ± 0.050 bits, *R*^2^ = 0.763 ± 0.024). Similarly, Fig. 4c, d show the G-P map parameters and GE measurement process inferred from toxicity measurements of TDP-43 variants, revealing among other things the toxicity-determining hot-spot observed by Bolognesi et al. [10] at positions 310–340. Again, the resulting latent phenotype model performs well on held-out test data (*I*_var_ = 1.834 ± 0.035 bits, *I*_pre_ = 1.994 ± 0.023 bits, *R*^2^ = 0.914 ± 0.007).

### Application: a massively parallel splicing assay

Exon/intron boundaries are defined by 5′ splice sites (5′ss), which bind the U1 snRNP during the initial stages of spliceosome assembly. To investigate how 5′ss sequence quantitatively controls alternative mRNA splicing, Wong et al. [11] used a massively parallel splicing assay (MPSA) to measure percent spliced in (PSI) values for nearly all 32,768 possible 5′ss of the form NNN/GYNNNN in three different genetic contexts (Fig. 1c,d). Applying MAVE-NN to data from the *BRCA2* exon 17 context, we inferred four different types of G-P maps: additive, neighbor, pairwise, and black box. As with GB1, these G-P maps were each inferred using GE regression with a heteroscedastic skewed-*t* noise model. For comparison, we also inferred an additive G-P map using the epistasis package of Sailer and Harms [28].

**Fig. 5:**
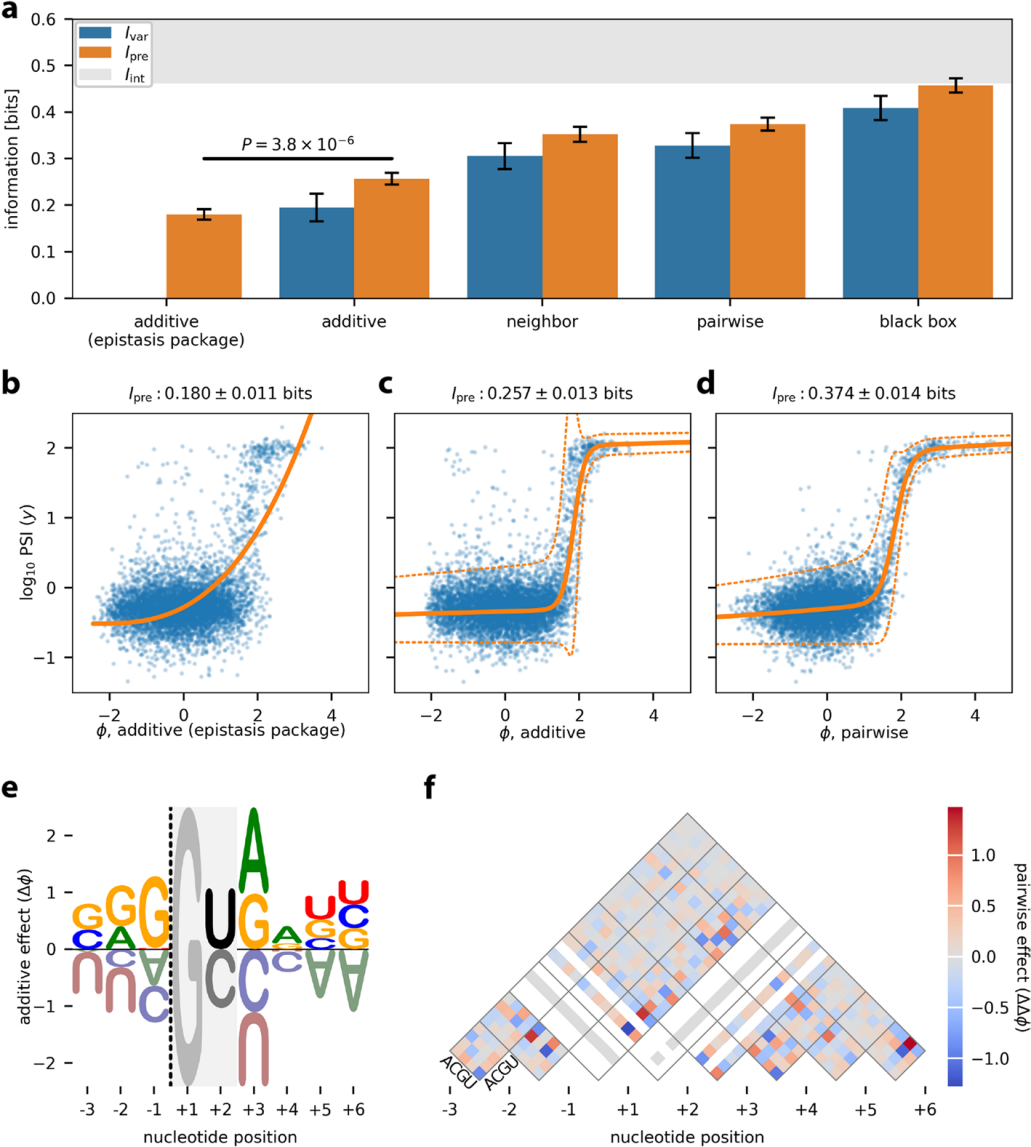
Analysis of MPSA data from Wong et al. [11]. This dataset reports PSI values, measured in the *BRCA2* exon 17 context, for nearly all 32,768 variant 5′ss of the form NNN/GYNNNN. Data were split 60:20:20 into training, validation, and test sets. Latent phenotype models were inferred, each comprising one of four types of G-P map (additive, neighbor, pairwise, or black box), together with a GE measurement process having a heteroscedastic skewed-*t* noise model. The epistasis package of Sailer and Harms [28] was also used to infer an additive G-P map and GE nonlinearity. **a** Performance of trained models as quantified by *I*_var_ and *I*_pre_, both computed on test data. The lower bound on *I*_int_ was estimated from experimental replicates (see “Methods”). The *p*-value reflects a two-sided *Z*-test. *I*_var_ was not computed for the additive (epistasis package) model because that package does not infer an explicit noise model. **b–d** Measurement values versus latent phenotype values, computed on test data, using the additive (epistasis package) model (**b**), the additive model (**c**), and the pairwise model (**d**). The corresponding GE measurement processes are also shown. **e** Sequence logo [40] illustrating the additive component of the pairwise G-P map. Dashed line indicates the exon/intron boundary. The G at +1 serves as a placeholder because no other bases were assayed at this position. At position +2, only U and C were assayed. **f** Heatmap showing the pairwise component of the pairwise G-P map. White diagonals correspond to unobserved bases. Additional file 1: Fig. S3 shows the uncertainties in the values of parameters that are illustrated in panels **e** and **f**. Error bars indicate standard errors. MPSA: massively parallel splicing assay; PSI: percent spliced in; G-P: genotype-phenotype; GE: global epistasis

Figure 5a compares the performance of these G-P map models on held-out test data, while Fig. 5b–d illustrate the corresponding inferred measurement processes. We observe that the additive G-P map inferred using the epistasis package [28] exhibits less predictive information (*I*_pre_ = 0.180 ± 0.011 bits) than the additive G-P map found using MAVE-NN (*P* = 3.8 × 10^−6^, two-sided *Z*-test). This is likely because the epistasis package estimates the parameters of the additive G-P map prior to estimating the GE nonlinearity. We also note that, while the epistasis package provides a variety of options for modeling the GE nonlinearity, none of these options appear to work as well as our more flexible sum-of-sigmoids approach (compare Fig. 5b,c). This finding again demonstrates that the accurate inference of G-P maps requires the explicit and simultaneous modeling of experimental nonlinearities.

We also observe that increasingly complex G-P maps exhibit increased accuracy. For example, the additive G-P map gives *I*_pre_ = 0.257 ± 0.013 bits, whereas the pairwise G-P map (Fig. 5d, f) attains *I*_pre_ = 0.374 ± 0.014 bits. Using MAVE-NN’s built-in parametric bootstrap approach for quantifying parameter uncertainty, we find that both the additive and pairwise G-P map parameters are very precisely determined (see Additional file 1: Fig. S3). The black box G-P map, which is comprised of 5 densely connected hidden layers of 10 nodes each, performed the best of all four G-P maps, achieving *I*_pre_ = 0.458 ± 0.015 bits. Remarkably, this predictive information reaches the lower bound on the intrinsic information estimated from replicate experiments (*I*_int_ ≥ 0.462 ± 0.009 bits; see “Methods”). The black box G-P map can, therefore, explain all of the apparent sequence-dependence in this MPSA dataset. We thus conclude that pairwise interaction models are not flexible enough to fully account for how 5′ss sequences control splicing. More generally, these results underscore the need for software that is capable of inferring and assessing a variety of different G-P maps through a uniform interface.

### Application: biophysically interpretable G-P maps

Biophysical models, unlike the phenomenological models considered thus far, have mathematical structures that reflect specific hypotheses about how sequence-dependent interactions between macromolecules mechanistically define G-P maps. Thermodynamic models, which rely on a quasi-equilibrium assumption, are the most commonly used type of biophysical model [41–43]. Previous studies have shown that precise thermodynamic models can be inferred from MAVE datasets [12, 44–46], but no software intended for use by the broader MAVE community has yet been developed for doing this. MAVE-NN meets this need by enabling the inference of custom G-P maps. We now demonstrate this biophysical modeling capability in the contexts of protein-ligand binding [using the DMS data of Olson et al. [8]; Fig. 1a(i)] and bacterial transcriptional regulation (using sort-seq MPRA data from Kinney et al. [12]; Fig. 1e). An expanded discussion of how these models are mathematically formulated and specified within MAVE-NN is provided in Additional file 1: Section S3.

**Fig. 6:**
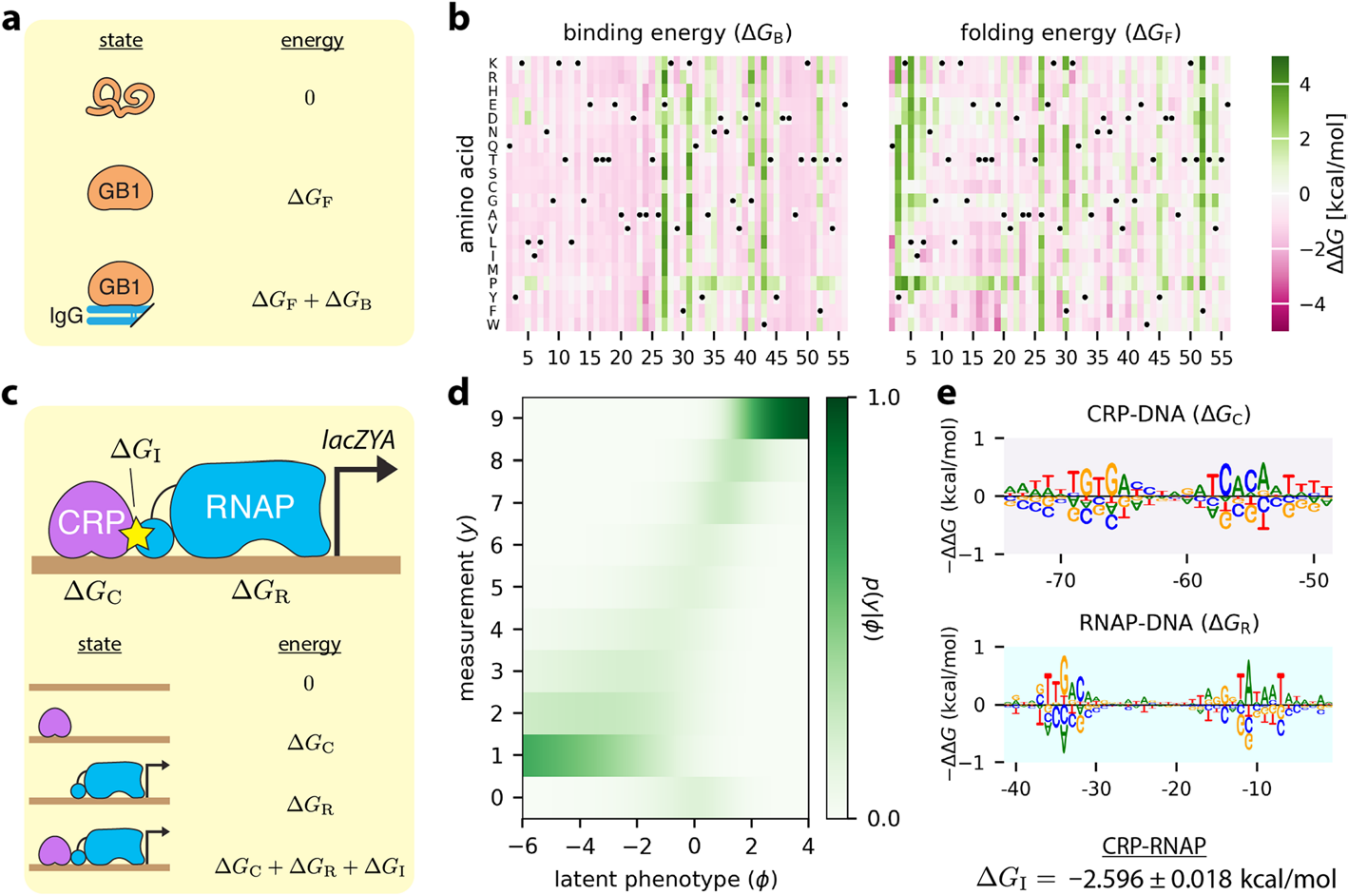
Biophysical models inferred from DMS and MPRA data. **a** Thermodynamic model for IgG binding by GB1. This model assumes that GB1 can be in one of three states (unfolded, folded-unbound, and folded-bound). The Gibbs free energies of folding (Δ*G*_F_) and binding (Δ*G*_B_) are computed from sequence using additive models, which in biophysical contexts like these are called energy matrices [1, 12]. The latent phenotype is given by the fraction of time GB1 is in the folded-bound state. **b** The ΔΔ*G* parameters of the energy matrices for folding and binding, inferred from the data of Olson et al. [8] by fitting this thermodynamic model using GE regression. The amino acid ordering used here matches that of Otwinowski [47]. Additional file 1: Fig. S5 plots folding energy predictions against the measurements of Nisthal et al. [49]. **c** A four-state thermodynamic model for transcriptional activation at the *E. coli lac* promoter. The Gibbs free energies of RNAP-DNA binding (Δ*G*_R_) and CRP-DNA binding (Δ*G*_C_) are computed using energy matrices, whereas the CRP-RNAP interaction energy Δ*G*_I_ is a scalar. The latent phenotype is the fraction of time a promoter is bound by RNAP. **d,e** The latent phenotype model inferred from the sort-seq MPRA data of Kinney et al. [12]. This model includes both the MPA measurement process (**d**) and the parameters of the thermodynamic G-P map (**e**). Additional file 1: Fig. S4 provides detailed definitions of the thermodynamic models in panels **a** and **c**. Sequence logos in panel **e** were generated using Logomaker [40]. Standard errors on Δ*G*_I_ were determined using the parametric bootstrap approach described in “Methods.” DMS: deep mutational scanning; MPRA: massively parallel reporter assay; IgG: immunoglobulin G; GB1: protein G domain B1; GE: global epistasis. RNAP: σ^70^ RNA polymerase; CRP: cAMP receptor protein; MPA: measurement process agnostic. G-P: genotype-phenotype

Otwinowski [47] showed that a three-state thermodynamic G-P map (Fig. 6a), one that accounts for GB1 folding energy in addition to GB1-IgG binding energy [48], can explain the DMS data of Olson et al. [8] better than a simple additive G-P map does. This biophysical model subsequently received impressive confirmation in the work of Nisthal et al. [49], who measured the thermostability of 812 single-mutation GB1 variants. We tested the ability of MAVE-NN to recover the same type of thermodynamic model that Otwinowski had inferred using custom analysis scripts. MAVE-NN yielded a G-P map with significantly improved performance on the data of Olson et al. [8] (*I*_var_ = 2.303 ± 0.013 bits, *I*_pre_ = 2.357 ± 0.007 bits, *R*^2^ = 0.947 ± 0.001) relative to the additive G-P map of Fig. 3a–d. Figure 6b shows the two inferred energy matrices that respectively describe the effects of every possible single-residue mutation on the Gibbs free energies of protein folding and protein-ligand binding. The folding energy predictions of our model also correlate as well with the data of Nisthal et al. [49] (*R*^2^ = 0.570 ± 0.049) as the predictions of Otwinowski’s model do (*R*^2^ = 0.515 ± 0.056). This demonstrates that MAVE-NN can infer accurate and interpretable quantitative models of protein biophysics.

To test MAVE-NN’s ability to infer thermodynamic models of transcriptional regulation, we re-analyzed the MPRA data of Kinney et al. [12], in which random mutations to a 75-bp region of the *Escherichia coli lac* promoter were assayed (Fig. 1e). This promoter region binds two regulatory proteins, σ^70^ RNA polymerase (RNAP) and the transcription factor CRP. As in Kinney et al. [12], we proposed a four-state thermodynamic model that quantitatively explains how promoter sequences control transcription rate (Fig. 6c). The parameters of this G-P map include the Gibbs free energy of interaction between CRP and RNAP (Δ*G*_I_), as well as energy matrices that describe the CRP-DNA and RNAP-DNA interaction energies (Δ*G*_C_ and Δ*G*_R_, respectively). Because the sort-seq MPRA of Kinney et al. [12] yielded discrete measurements (Fig. 1f), we used an MPA measurement process in our latent phenotype model (Fig. 6d). The biophysical parameters we thus inferred (Fig. 6e), which include a CRP-RNAP interaction energy of Δ*G*_I_ =  − 2.598 ± 0.018 kcal/mol, largely match those of Kinney et al., but were obtained far more rapidly (in ~10 min versus multiple days) thanks to the use of stochastic gradient descent rather than Metropolis Monte Carlo.

### Constraints on datasets and models

As mentioned above, MAVE-NN places certain limitations on both input datasets and latent phenotype models. Some of these constraints have been adopted to simplify the initial release of MAVE-NN and can be relaxed in future updates. Others reflect fundamental mathematical properties of latent phenotype models. Here we summarize the primary constraints users should be aware of.

MAVE-NN currently requires that all input sequences be the same length. This constraint has been adopted because a large fraction of MAVE datasets have this form, and all of the built-in G-P maps operate only on fixed-length sequences. Users who wish to analyze variable length sequences can still do so by padding the ends of sequences with dummy characters. Alternatively, users can provide a multiple-sequence alignment as input and include the gap character as one of the characters to consider when training models.

As previously stated, MAVE-NN can analyze MAVE datasets that have either continuous or discrete measurements. At present, both types of measurements must be one-dimensional, i.e., users cannot fit a single model to vectors of multiple measurements (e.g., joint measurements of protein binding affinity and protein stability, as in Faure et al. [31]). This constraint has been adopted only to simplify the user interface of the initial release. It is not a fundamental limitation of latent phenotype models and is scheduled to be relaxed in upcoming versions of MAVE-NN.

The current implementation of MAVE-NN also supports only one-dimensional latent phenotypes (though the latent phenotype of custom G-P maps can depend on multiple precursor phenotypes, e.g., binding energy or folding energy). This restriction was made because accurately interpreting multi-dimensional latent phenotypes is substantially more fraught than interpreting one-dimensional latent phenotypes, and we believe that additional computational tools need to be developed to facilitate such interpretation. That being said, the mathematical form of latent phenotype models is fully compatible with multi-dimensional latent phenotypes. Indeed, this modeling strategy has been used in other work [24, 31–33], and we plan to enable this functionality in future updates to MAVE-NN.

More fundamental constraints come into play when analyzing MAVE data that contains only single-mutation variants. In such experiments, the underlying effects of individual mutations are hopelessly confounded by the biophysical, physiological, and experimental nonlinearities that may be present. By contrast, when the same mutation is observed in multiple genetic backgrounds, MAVE-NN can use systematic differences in the mutational effects observed between stronger and weaker backgrounds to remove these confounding influences. Thus, for datasets that probe only single-mutant effects, we limit MAVE-NN to inferring only additive G-P maps using GE regression, and while the noise model in the GE measurement process is allowed to be heteroscedastic, the nonlinearity is constrained to be linear.

We emphasize that, in practice, only a modest number of multiple-mutation variants are required for MAVE-NN to learn the form of a nonlinear measurement process (see Fig. 3e–g). In this way, including a small fraction of the possible double-mutation variants in MAVE libraries can be beneficial even just for determining the effects of single mutations. Adding such non-comprehensive sets of double mutants to MAVE libraries is experimentally straight-forward, and our numerical experiments suggest that assaying roughly the same number of double-mutation variants as single-mutation variants should often suffice. We therefore recommend that experimentalists—even those primarily interested in the effects of single mutations—consider augmenting their MAVE libraries with a small subset of double-mutation variants.

## Discussion

We have presented a unified strategy for inferring quantitative models of G-P maps from diverse MAVE datasets. At the core of our approach is the conceptualization of G-P maps as a form of information compression, i.e., the G-P map first compresses an input sequence into a latent phenotype value, which is then read out indirectly via a noisy and nonlinear measurement process. By explicitly modeling this measurement process, one can remove potentially confounding effects from the G-P map, as well as accommodate diverse experimental designs. We have also introduced three information-theoretic metrics for assessing the performance of the resulting models. These capabilities have been implemented within an easy-to-use Python package called MAVE-NN.

We have demonstrated the capabilities of MAVE-NN in diverse biological contexts, including in the analysis of both DMS and MPRA data. We have also demonstrated the superior performance of MAVE-NN relative to the epistasis package of Sailer and Harms [28]. Along the way, we observed that MAVE-NN can deconvolve experimental nonlinearities from additive G-P maps when a relatively small number of sequences containing multiple mutations are assayed. This capability provides a compelling reason for experimentalists to include such sequences in their MAVE libraries, even if they are primarily interested in the effects of single mutations. Finally, we showed how MAVE-NN can learn biophysically interpretable G-P maps from both DMS and MPRA data.

MAVE-NN thus fills a critical need in the MAVE community, providing user-friendly software capable of learning quantitative models of G-P maps from diverse MAVE datasets. MAVE-NN has a streamlined user interface and is readily installed from PyPI by executing “pip install mavenn” at the command line. Comprehensive documentation and step-by-step tutorials are available at http://mavenn.readthedocs.io [50].

## Methods

### Notation

We represent each MAVE dataset as a set of *N* observations, ${\left\{\left({x}_n,{y}_n\right)\right\}}_{n=0}^{N-1}$, where each observation consists of a sequence *x*_*n*_ and a measurement *y*_*n*_. Here, *y*_*n*_ can be either a continuous real-valued number, or a nonnegative integer representing a “bin” in which the *n*th sequence was found. Note that, in this representation, the same sequence *x* can be observed multiple times, potentially with different values for *y* due to experimental noise.

### G-P maps

We assume that all sequences have the same length *L* and that at each of the *L* positions in each sequence there is one of *C* possible characters. MAVE-NN represents sequences using a vector of one-hot encoded features of the form

1 $${x}_{l:c}\kern0.5em =\kern0.5em \left\{\begin{array}{cl}\ 1& \mathrm{if}\ \mathrm{character}\ c\ \mathrm{occurs}\ \mathrm{at}\ \mathrm{position}\ l\\ {}\ 0& \mathrm{otherwise}\end{array}\right.,$$

where *l* = 0, 1, …, *L* − 1 indexes positions within the sequence, and *c* indexes the *C* distinct characters in the alphabet. MAVE-NN supplies built-in alphabets for DNA, RNA, and protein (with or without stop codons) and supports custom alphabets as well.

We assume that the latent phenotype is given by a linear function *ϕ*(*x*; *θ*) that depends on a set of G-P map parameters *θ*. As mentioned in the main text, MAVE-NN supports four types of G-P map models, all of which can be inferred using either GE regression or MPA regression. The additive model is given by

2 $${\phi}_{\mathrm{additive}}\left(x;\theta \right) = {\theta}_0+\sum_{l=0}^{L-1}\sum_c{\theta}_{l:c}\,{x}_{l:c}.$$

Here, each position in *x* contributes independently to the latent phenotype. The neighbor model is given by

3 $${\phi}_{\mathrm{neighbor}}\left(x;\theta \right)={\theta}_0+\sum_{l=0}^{L-1}\sum_c{\theta}_{l:c}\,{x}_{l:c}+\sum_{l=0}^{L-2}\sum_{c,{c}^{\prime }}{\theta}_{l:c,(l+1):{c}^{\prime }}\,{x}_{l:c}\,{x}_{(l+1):{c}^{\prime }},$$

and further accounts for potential epistatic interactions between neighboring positions. The pairwise model is given by

4 $${\phi}_{\mathrm{pairwise}}\left(x;\theta \right)={\theta}_0+\sum_{l=0}^{L-1}\sum_c{\theta}_{l:c}\,{x}_{l:c}+\sum_{l=0}^{L-2}\sum_{l^{\prime }=l+1}^{L-1}\sum_{c,{c}^{\prime }}{\theta}_{l:c,{l}^{\prime }:{c}^{\prime }}\,{x}_{l:c}\,{x}_{l^{\prime }:{c}^{\prime }},$$

and includes interactions between all pairs of positions. Note our convention of requiring *l* ′  > *l* in the pairwise parameters ${\theta}_{l:c,{l}^{\prime }:{c}^{\prime }}$.

Unlike these three parametric models, the black box G-P map does not have a fixed functional form. Rather, it is given by a multilayer perceptron that takes a vector of sequence features (additive, neighbor, or pairwise) as input, contains multiple fully connected hidden layers with nonlinear activations, and has a single node output with a linear activation. Users are able to specify the number of hidden layers, the number of nodes in each hidden layer, and the activation function used by these nodes.

MAVE-NN further supports custom G-P maps, which users can define by subclassing the G-P map base class. These G-P maps can have arbitrary functional forms, e.g., representing specific biophysical hypotheses. This feature of MAVE-NN is showcased in Fig. 6.

### Gauge modes and diffeomorphic modes

G-P maps typically have non-identifiable degrees of freedom that must be fixed, i.e., pinned down, before the values of individual parameters can be meaningfully interpreted or compared between models. These degrees of freedom come in two flavors: gauge modes and diffeomorphic modes. Gauge modes are changes to *θ* that do not alter the values of the latent phenotype *ϕ*. Diffeomorphic modes [20, 24] are changes to *θ* that do alter *ϕ*, but do so in ways that can be undone by transformations of the measurement process *p*(*y*| *ϕ*). As shown by Kinney and Atwal [20, 24], the diffeomorphic modes of linear G-P maps (such as the additive and pairwise G-P maps featured in Figs. 3, 4, and 5) will typically correspond to affine transformations of *ϕ*, although additional unconstrained modes can occur in special situations.

MAVE-NN automatically fixes the gauge modes and diffeomorphic modes of inferred models (except when using custom G-P maps). The diffeomorphic modes of G-P maps are fixed by transforming *θ* via

5 $${\theta}_0\to {\theta}_0-a,$$

and then

6 $$\theta \to \frac{\theta }{b},$$

where *a* = mean({*ϕ*_*n*_}) and *b* = std({*ϕ*_*n*_}) are the mean and standard deviation of *ϕ* values computed on the training data. This produces a corresponding change in latent phenotype values *ϕ* → (*ϕ* − *a*)/*b*. To avoid altering model likelihood, MAVE-NN makes a corresponding transformation to the measurement process *p*(*y*| *ϕ*). In GE regression this is done by adjusting the GE nonlinearity via

7 $$g\left(\phi \right)\to g\left(a+ b\phi \right),$$

while keeping the noise model $p\left(y|\hat{y}\right)$ fixed. In MPA regression, MAVE-NN transforms the full measurement process via

8 $${\displaystyle \begin{array}{r}p\left(y|\phi \right)\to p\left(y|a+ b\phi \right).\end{array}}$$

For the three parametric G-P maps, gauge modes are fixed using what we call the “hierarchical gauge”. Here, the parameters *θ* are adjusted so that the lower-order terms in *ϕ*(*x*; *θ*) account for the highest possible fraction of variance in *ϕ*. This procedure requires a probability distribution on sequence space with respect to which these variances are computed. MAVE-NN assumes that such distributions factorize by position and can thus be represented by a probability matrix with elements *p*_*l* : *c*_, denoting the probability of character *c* at position *l*. MAVE-NN provides three built-in choices for this distribution: uniform, empirical, or wildtype. The corresponding values of *p*_*l* : *c*_ are given by

9 $${\displaystyle \begin{array}{rrr}{p}_{l:c}& =& \left\{\begin{array}{cc}1/C& \mathrm{for}\ \mathrm{uniform}\\ {}{n}_{l:c}/N& \mathrm{for}\ \mathrm{empirical}\\ {}{x}_{l:c}^{\mathrm{WT}}& \mathrm{for}\ \mathrm{wildtype}\end{array}\right.,\end{array}}$$

where *n*_*l* : *c*_ denotes the number of observations in the dataset (out of *N* total) for which the sequence has character *c* at position *l*, and ${x}_{l:c}^{\mathrm{WT}}$ is the one-hot encoding of a user-specified wildtype sequence. In particular, the wildtype gauge is used for illustrating the additive G-P maps in Figs. 3 and 4, while the uniform gauge is used for illustrating the pairwise G-P map in Fig. 5 and the energy matrices in Fig. 6. After a sequence distribution is chosen, MAVE-NN fixes the gauge of the pairwise G-P map by transforming

10 $$\begin{aligned} {\theta}_{0} \to &{\theta}_{0} \\& +\sum_l\sum_{c^{\prime }}{\theta}_{l:{c}^{\prime }}\,{p}_{l:{c}^{\prime }}\ \\& +\sum_l\sum_{l^{\prime }>l}\sum_{c,{c}^{\prime }}{\theta}_{l:c,{l}^{\prime }:{c}^{\prime }}\,{p}_{l:c}\,{p}_{l^{\prime }:{c}^{\prime }},\end{aligned}$$

11 $$\begin{aligned} \theta_{l:c} \to &{\theta}_{l:c}\ \\& -\sum_{c^{\prime }}{\theta}_{l:{c}^{\prime }}\,{p}_{l:{c}^{\prime }}\ \\& +\sum_{l^{\prime }>l}\sum_{c^{\prime }}{\theta}_{l:c,{l}^{\prime }:{c}^{\prime }}\,{p}_{l^{\prime }:{c}^{\prime }}\ \\& +\sum_{l^{\prime }<l}\sum_{c^{\prime }}{\theta}_{l^{\prime }:{c}^{\prime },l:c}\,{p}_{l^{\prime }:{c}^{\prime }}\ \\& -\sum_{l^{\prime }>l}\sum_{c^{\prime },{c}^{{\prime\prime} }}{\theta}_{l:{c}^{\prime },{l}^{\prime }:{c}^{{\prime\prime} }}\,{p}_{l:{c}^{\prime }}\,{p}_{l^{\prime }:{c}^{{\prime\prime} }}\ \\& -\sum_{l^{\prime }<l}\sum_{c^{\prime },{c}^{{\prime\prime} }}{\theta}_{l^{\prime}:{c}^{{\prime\prime} },l:{c}^{\prime }}\,{p}_{l:{c}^{\prime }}\,{p}_{l^{\prime }:{c}^{{\prime\prime} }} ,\end{aligned}$$

and

12 $$\begin{aligned} {\theta}_{l:c,{l}^{\prime}:{c}^{\prime}} \to & {\theta}_{l:c,{l}^{\prime }:{c}^{\prime }}\\& -\sum_{c^{{\prime\prime} }}{\theta}_{l:{c}^{{\prime\prime} },{l}^{\prime }:{c}^{\prime }}{p}_{l:{c}^{{\prime\prime} }}\\& -\sum_{c^{{\prime\prime} }}{\theta}_{l:c,{l}^{\prime }:{c}^{{\prime\prime} }}{p}_{l^{\prime }:{c}^{{\prime\prime} }}\\& +\sum_{c^{{\prime\prime} },{c}^{{\prime\prime\prime} }}{\theta}_{l:{c}^{{\prime\prime} },{l}^{\prime }:{c}^{{\prime\prime\prime} }}\;{p}_{l:{c}^{{\prime\prime} }}\;{p}_{l^{\prime }:{c}^{{\prime\prime\prime} }}.\end{aligned}$$

This transformation is also used to gauge-fix the additive and neighbor G-P maps, but with ${\theta}_{l:c,{l}^{\prime }:{c}^{\prime }}=0$ for all *l*, *l*′ (additive model), or when *l* ′  ≠ *l* + 1 (neighbor model).

### GE nonlinearities

GE models assume that each measurement *y* is a nonlinear function *g*(·) of the latent phenotype *ϕ*, plus some noise. In MAVE-NN, this nonlinearity is represented as a sum of hyperbolic tangent sigmoids:

13 $$g\left(\phi; \alpha \right)=a+\sum_{k=0}^{K-1}{b}_k\tanh \left({c}_k\phi +{d}_k\right).$$

Here, *K* specifies the number of hidden nodes contributing to the sum, and *α* = {*a*, *b*_*k*_, *c*_*k*_, *d*_*k*_} are trainable parameters. We note that this mathematical form is an example of the bottleneck architecture previously used by others [27, 33] for modeling GE nonlinearities. By default, MAVE-NN constrains *g*(*ϕ*; *α*) to be monotonic in *ϕ* by requiring all *b*_*k*_ ≥ 0 and *c*_*k*_ ≥ 0, but this constraint can be relaxed.

### GE noise models

MAVE-NN supports three types of GE noise model: Gaussian, Cauchy, and skewed-*t*. All of these noise models support the analytic computation of quantiles and prediction intervals, as well as the rapid sampling of simulated measurement values. The Gaussian noise model is given by

14 $${p}_{\mathrm{gauss}}\left(y|\hat{y};s\right)=\frac{1}{\sqrt{2\pi {s}^2}}\exp \left[-\frac{{\left(y-\hat{y}\right)}^2}{2{s}^2}\right],$$

where *s* denotes the standard deviation. Importantly, MAVE-NN allows this noise model to be heteroscedastic by representing *s* as an exponentiated polynomial in $\hat{y}$, i.e.,

15 $$s\left(\hat{y}\right)=\exp \left[\sum_{k=0}^K{a}_k{\hat{y}}^k\right],$$

where *K* is the order of the polynomial and {*a*_*k*_} are trainable parameters. The user has the option to set *K*, and setting *K* = 0 renders this noise model homoscedastic. Quantiles are computed using ${y}_q=\hat{y}+s\;\sqrt{2}\;{\operatorname{erf}}^{-1}\left(2q-1\right)$ for user-specified values of *q* ∈ [0, 1]. Similarly, the Cauchy noise model is given by

16 $${p}_{\mathrm{cauchy}}\left(y|\hat{y};s\right)={\left[\pi s\left(1+\frac{{\left(y-\hat{y}\right)}^2}{s^2}\right)\right]}^{-1},$$

where the scale parameter *s* is an exponentiated *K*-order polynomial in $\hat{y}$, and quantiles are computed using ${y}_q=\hat{y}+s\;\tan \left[\pi \left(q-\frac{1}{2}\right)\right]$.

The skewed-*t* noise model is of the form described by Jones and Faddy [34] and is given by

17 $${p}_{\mathrm{skewt}}\left(y|\hat{y};s,a,b\right)={s}^{-1}f\left(t;a,b\right),$$

where

18 $$t={t}^{\ast }+\frac{y-\hat{y}}{s},\ \ {t}^{\ast }=\frac{\left(a-b\right)\sqrt{a+b}}{\sqrt{2a+1}\sqrt{2b+1}},$$

and

19 $$f\left(t;a,b\right)= \frac{2^{1-a-b}}{\sqrt{a+b}}\frac{\Gamma \left(a+b\right)}{\Gamma (a)\Gamma (b)}\ {\left[1+\frac{t}{\sqrt{a+b+{t}^2}}\right]}^{a+\frac{1}{2}}\times {\left[1-\frac{t}{\sqrt{a+b+{t}^2}}\right]}^{b+\frac{1}{2}}.$$

Note that the *t* statistic here is an affine function of *y* chosen so that the distribution’s mode (corresponding to *t*^∗^) is positioned at $\hat{y}$. The three parameters of this noise model, {*s*, *a*, *b*}, are each represented using *K*-order exponentiated polynomials with trainable coefficients. Quantiles are computed using

20 $${y}_q=\hat{y}+\left({t}_q-{t}^{\ast}\right)s,$$

where

21 $${t}_q=\frac{\left(2{x}_q-1\right)\sqrt{a+b}}{\sqrt{1-{\left(2{x}_q-1\right)}^2}},\ \ {x}_q={I}_q^{-1}\left(a,b\right),$$

and ${I}_q^{-1}\left(a,b\right)$ denotes the inverse of the regularized incomplete Beta function *I*_*x*_(*a*, *b*).

### Empirical noise models

MAVE-NN further supports the inference of GE regression models that account for user-specified measurement noise. In such cases, the user provides a set of measurement-specific standard errors, ${\left\{{s}_n\right\}}_{n=0}^{N-1}$, along with the corresponding observations. These uncertainties can, for example, be estimated by using a software package like Enrich2 [16] or DiMSum [19]. MAVE-NN then trains the parameters of latent phenotype models by assuming a Gaussian noise model of the form

22 $${p}_{\mathrm{empirical}}\left({y}_n|{\hat{y}}_n,{s}_n\right)=\frac{1}{\sqrt{2\pi {s}_n^2}}\ \exp \left[-\frac{{\left({y}_n-{\hat{y}}_n\right)}^2}{2{s}_n^2}\right],$$

where ${\hat{y}}_n=g\left(f\left({x}_n;\theta \right);\alpha \right)$ is the expected measurement for sequence *x*_*n*_, *θ* denotes G-P map parameters, and *α* denotes the parameters of the GE nonlinearity. This noise model thus has the advantage of having no free parameters, but it may be problematically misspecified if the true error distribution is heavy-tailed or skewed.

### MPA measurement process

In MPA regression, MAVE-NN directly models the measurement process *p*(*y*| *ϕ*). At present, MAVE-NN only supports MPA regression for discrete values of *y*, which must be indexed using nonnegative integers. MAVE-NN supports two alternative forms of input for MPA regression. One is a set of sequence-measurement pairs, ${\left\{\left({x}_n,{y}_n\right)\right\}}_{n=0}^{N-1}$, where *N* is the total number of reads, {*x*_*n*_} is a set of (typically non-unique) sequences, each *y*_*n*_ ∈ {0, 1, …, *Y* − 1} is a bin number, and *Y* is the total number of bins. The other is a set of sequence-count-vector pairs, ${\left\{\left({x}_m,{c}_m\right)\right\}}_{m=0}^{M-1}$, where *M* is the total number of unique sequences and *c*_*m*_ = (*c*_*m*0_, *c*_*m*1_, …, *c*_*m*(*Y* − 1)_) is a vector that lists the number of times *c*_*my*_ that the sequence *x*_*m*_ was observed in each bin *y*. MPA measurement processes are represented as a multilayer perceptron with one hidden layer (having tanh activations) and a softmax output layer. Specifically,

23 $$p\left(y|\phi \right)=\frac{w_y\left(\phi \right)}{\sum_{y^{\prime }}{w}_{y^{\prime }}\left(\phi \right)},$$

where

24 $${w}_y\left(\phi \right)=\exp \left[{a}_y+\sum_{k=0}^{K-1}{b}_{yk}\tanh \left({c}_{yk}\phi +{d}_{yk}\right)\right],$$

and *K* is the number of hidden nodes per value of *y*. The trainable parameters of this measurement process are *η* = {*a*_*y*_, *b*_*yk*_, *c*_*yk*_, *d*_*yk*_}.

### Loss function

Let *θ* denote the G-P map parameters, and *η* denote the parameters of the measurement process. MAVE-NN optimizes these parameters using stochastic gradient descent on a loss function given by

25 $$\mathcal{L}={\mathcal{L}}_{\mathrm{like}}+{\mathcal{L}}_{\mathrm{reg}},$$

where ${\mathcal{L}}_{\mathrm{like}}$ is the negative log likelihood of the model, given by

26 $${\mathcal{L}}_{\mathrm{like}}\left[\theta, \eta \right]=-\sum_{n=0}^{N-1}\log \left[p\left({y}_n|{\phi}_n;\eta \right)\right],$$

where *ϕ*_*n*_ = *ϕ*(*x*_*n*_; *θ*),and ${\mathcal{L}}_{\mathrm{reg}}$ provides for the regularization of both *θ* and *η*.

In the context of GE regression, we can write *η* = (*α*, *β*) where *α* represents the parameters of the GE nonlinearity *g*(*ϕ*; *α*) and *β* denotes the parameters of the noise model $p\left(y|\hat{y};\beta \right)$. The likelihood contribution from each observation *n* then becomes $p\left({y}_n|{\phi}_n;\eta \right)=p\left({y}_n|{\hat{y}}_n;\beta \right)$ where ${\hat{y}}_n=g\left({\phi}_n;\alpha \right)$. In the context of MPA regression with a dataset of the form ${\left\{\left({x}_m,{c}_m\right)\right\}}_{m=0}^{M-1}$, the loss function can be written as

27 $${\mathcal{L}}_{\mathrm{like}}\left[\theta, \eta \right]=-\sum_{m=0}^{M-1}\sum_{y=0}^{Y-1}{c}_{my}\log \left[p\left(y|{\phi}_m;\eta \right)\right]$$

where *ϕ*_*m*_ = *ϕ*(*x*_*m*_; *θ*). For the regularization term, MAVE-NN uses an *L*_2_ penalty of the form

28 $${\mathcal{L}}_{\mathrm{reg}}\left[\theta, \eta \right]={\lambda}_{\theta }{\left\Vert \theta \right\Vert}^2+{\lambda}_{\eta }{\left\Vert \eta \right\Vert}^2,$$

where the user-adjustable parameters *λ*_*θ*_ (default value 10^−3^) and *λ*_*η*_ (default value 10^−1^) respectively control the strength of regularization for *θ* and *η*.

### Predictive information

In what follows, we use *p*_model_(*y*| *ϕ*) to denote a measurement process inferred by MAVE-NN, whereas *p*_true_(*y*| *ϕ*) denotes the empirical conditional distribution of *y* and *ϕ* values that would be observed in the limit of infinite test data. Predictive information is defined by *I*_pre_ = *I*[*y*; *ϕ*], where *I*[·; ·] represents mutual information computed on data not used for training (i.e., a held-out test set or data from a different experiment). *I*_pre_ provides a measure of how strongly a G-P map predicts experimental measurements. Importantly, this quantity does not depend on the corresponding measurement process *p*_model_(*y*| *ϕ*). To estimate *I*_pre_, we use *k*-nearest neighbor (kNN) estimators of entropy and mutual information adapted from the NPEET Python package [51]. Here, the user has the option of adjusting *k*, which controls a variance/bias tradeoff. When *y* is discrete (MPA regression), *I*_pre_ is computed using the classic kNN entropy estimator [52, 53] via the decomposition $I\left[y;\phi \right]=H\left[\phi \right]-\sum_yp(y){H}_y\left[\phi \right]$, where *H*_*y*_[*ϕ*] denotes the entropy of *p*_true_(*ϕ*| *y*). When *y* is continuous (GE regression), *I*[*y*; *ϕ*] is estimated using the kNN-based Kraskov Stögbauer Grassberger (KSG) algorithm [53]. This approach optionally supports the local nonuniformity correction of Gao et al. [54], which is important when *y* and *ϕ* exhibit strong dependencies, but which also requires substantially more time to compute.

### Variational information

We define variational information as an affine transformation of ${\mathcal{L}}_{\mathrm{like}}$,

29 $${I}_{\mathrm{var}}=H\left[y\right]-\frac{\log_2(e)}{N}{\mathcal{L}}_{\mathrm{like}}.$$

Here, *H*[*y*] is the entropy of the data {*y*_*n*_}, which is estimated using the kNN estimator from the NPEET package [51]. Noting that this quantity can also be written as *I*_var_ = *H*[*y*] − mean({*Q*_*n*_}), where *Q*_*n*_ =  − log_2_*p*(*y*_*n*_| *ϕ*_*n*_), we estimate the associated uncertainty (denoted by *δ*) using

30 $$\delta {I}_{\mathrm{var}}\left[y;\phi \right]=\sqrt{\delta H{\left[y\right]}^2+\frac{\operatorname{var}\left(\left\{{Q}_n\right\}\right)}{N}}.$$

The inference strategy used by MAVE-NN is based on the fact that *I*_var_ provides a tight variational lower bound on *I*_pre_. Indeed, in the large data limit,

31 $$I_{\text{pre}}=I_{\text{var}}+D_{\text{KL}}\left(p_{\text{true}}\parallel p_{\text{model}}\right),$$

where *D*_KL_(·) ≥ 0 is a Kullback-Leibler divergence that quantifies the accuracy of the inferred measurement process. From Eq. 31 one can see that, with appropriate caveats, maximizing *I*_var_ (or equivalently, minimizing ${\mathcal{L}}_{\mathrm{like}}$) will also maximize *I*_pre_ [24]. But unlike *I*_pre_, *I*_var_ is readily compatible with backpropagation and stochastic gradient descent. See Additional file 1 for a derivation of Eq. 31 and a discussion of relevant prior work [20, 24, 37–39]. We note that Sharpee et al. [55] cleverly showed that *I*_pre_ can, in fact, be optimized using stochastic gradient descent. Computing gradients of *I*_pre_, however, requires a time-consuming density estimation step, whereas optimizing *I*_var_ can be done using standard per-datum backpropagation.

### Intrinsic information

Intrinsic information, *I*_int_ = *I*[*x*; *y*], is the mutual information between the sequences *x* and measurements *y* in a dataset. This quantity is somewhat tricky to estimate due to the high-dimensional nature of sequence space. Here we used three different methods to obtain the upper and lower bounds on *I*_int_ shown in Figs. 3d and 5a. To compute the upper bound on *I*_int_ for the GB1 data of Olson et al. [8] (in Fig. 3d), we used the fact that

32 $$I\left[x;y\right]=H\left[y\right]-{\left\langle {H}_x\left[y\right]\right\rangle}_x,$$

where *H*[*y*] is the entropy of all measurements *y*, *H*_*x*_[*y*] is the entropy of *p*(*y*| *x*) for a specific choice of sequence *x*, and ${\left\langle\cdot\right\rangle}_x$ indicates averaging over the sequences *x* in the dataset. Here, the measurement value *y*_*n*_ for each sequence *x* was computed using Eq. 36 (below). *H*[*y*] was then estimated using the kNN estimator applied to these measurements [52]. We also estimated the uncertainty in *y*_*n*_ by propagating errors expected due to Poisson fluctuations in read counts:

33 $$\delta {y}_n={\log}_2(e)\sqrt{\frac{1}{c_n^{\mathrm{in}}+1}+\frac{1}{c_n^{\mathrm{out}}+1}}.$$

Assuming *p*(*y*| *x*) to be Gaussian, we find the corresponding conditional entropy to be

34 $${H}_{x_n}\left[{y}_n\right]=\frac{1}{2}{\log}_2\left(2\pi e\;\delta {y}_n^2\right).$$

Notice that we did not include the effects of fluctuations in ${c}_{\mathrm{WT}}^{\mathrm{in}}$ or ${c}_{\mathrm{WT}}^{\mathrm{out}}$ in Eq. 33, as these shift all *y* values by the same amount and thus do not affect either *H*[*y*] or *H*_*x*_[*y*].

These *H*[*y*] and *H*_*x*_[*y*] values were then used in Eq. 32 to estimate *I*_int_. We expect this to provide an upper bound on the true value of *I*_int_ because uncertainty in *y* must be at least that expected under the Poisson sampling of reads. However the use of linear error propagation and the assumption that *p*(*y*| *x*) is approximately Gaussian complicate this conclusion. Also, when applied to MPSA data, this method yielded an upper bound of 0.96 bits, a value that is likely far higher than the true value of *I*_int_. This mismatch likely resulted from read counts in the MPSA data being over-dispersed.

To compute the lower bound on *I*_int_ for the GB1 dataset (Fig. 3d), we used the predictive information *I*_pre_ (on test data) of a GE regression model having a black box G-P map. This provides a lower bound because *I*_int_ ≥ *I*_pre_ for any model (when evaluated on test data) due to the Data Processing Inequality and the Markov chain nature of the dependencies *y* ← *x* → *ϕ* in Fig. 2e [24, 36].

To compute a lower bound on *I*_int_ for MPSA data (Fig. 5c), we leveraged the availability of replicate data in Wong et al. [11]. Let *y* and *y*′ represent the original and replicate measurements obtained for a sequence *x*. Because *y* ← *x* → *y*′ forms a Markov chain, *I*[*x*; *y*] ≥ *I*[*y*; *y*′] [36]. We therefore used an estimate of *I*[*y*; *y*′], computed using the KSG method [51, 53], as the lower bound for *I*_int_.

### Uncertainties in kNN estimates of mutual information

MAVE-NN quantifies uncertainties in *H*[*y*] and *I*[*y*; *ϕ*] using multiple random samples of half the data. Let ${\mathcal{D}}_{100\%}$ denote the dataset on which we wish to compute these quantities, and let ${\mathcal{D}}_{50\%,r}$ denote a 50% subsample (indexed by *r*) of this dataset. Given an estimator *E*(·) of either entropy or mutual information, as well as the number of subsamples *R* to use, the uncertainty in $E\left({\mathcal{D}}_{100\%}\right)$ is estimated as

35 $$\delta E\left({\mathcal{D}}_{100\%}\right)=\frac{1}{\sqrt{2}}\mathrm{std}\left[{\left\{E\left({\mathcal{D}}_{50\%,r}\right)\right\}}_{r=0}^{R-1}\right].$$

MAVE-NN uses *R* = 25 by default. Note that computing such uncertainty estimates substantially increases computation time, as *E*(·) needs to be evaluated *R* + 1 times instead of just once. We also note that we favor this subsampling approach, as opposed to standard bootstrap resampling [56, 57], as the latter can systematically underestimate *H*[*y*] and overestimate *I*[*y*; *z*].

### Uncertainties in G-P map parameters

Given a trained latent phenotype model having G-P map parameters *θ*^∗^ and measurement process parameters *η*^∗^, MAVE-NN can optionally assess model uncertainty using the following parametric bootstrap approach. Using the trained model with parameters (*θ*^∗^, *η*^∗^) as “ground truth,” MAVE-NN first simulates *R* MAVE datasets ${\mathcal{D}}_r={\left\{\left({x}_n,{y}_n^{(r)}\right)\right\}}_{n=0}^{N-1}$, where *r* = 0, 1, …, *R* − 1. For each simulated dataset ${\mathcal{D}}_r$, MAVE-NN then trains a new model (by default using the same hyperparameters as were used for the ground truth model). This procedure yields a set ${\left\{\left({\theta}^{(r)},{\eta}^{(r)}\right)\right\}}_{r=0}^{R-1}$ of simulation-inferred G-P map parameters and corresponding measurement process parameters. Users can then use this sampling of G-P map parameters to estimate uncertainties, e.g., by reporting $\delta {\theta}_k=\mathrm{std}\left[{\left\{{\theta}_k^{(r)}\right\}}_{r=0}^{R-1}\right]$.

There are good reasons to estimate parameter uncertainties using this parametric bootstrap approach, rather than through other bootstrapping methods. In particular, the parametric bootstrap keeps the sequences in each simulated data set fixed, and treats only the measurements as being stochastic. This comports with the fact that, in many MAVE designs, a specific set of user-specified sequences are assayed, with each sequence receiving a single measurement value. If each simulated dataset were instead generated by resampling sequence-measurement pairs from the full dataset, virtually all of these simulated datasets would end up lacking measurements for a substantial subset of the original assayed sequences. Among other issues, this can lead to parameter non-identifiability in models trained on simulated data even when such non-identifiability is absent in models trained on the original dataset.

Another important detail when assessing parameter uncertainty is to ensure that both the gauge modes and the diffeomorphic modes of each model are fixed. This is necessary so that differences in the parameters that do not affect model predictions do not inflate uncertainty estimates. For additive, neighbor, and pairwise G-P maps, MAVE-NN automatically implements the procedure described in the “Gauge modes and diffeomorphic modes” section above, thereby removing these extra degrees of freedom. However, for more complex models such as those implemented by MAVE-NN’s custom G-P map functionality (e.g., representing biophysical models), different gauge freedoms and diffeomorphic modes may arise depending on the details of the model, and users must take care to determine and fix these prior to assessing parameter uncertainty. We also note that no meaningful computation of individual parameter uncertainties is likely to be possible for highly overparameterized models, such as the “black box” multilayer perceptron models supported by MAVE-NN.

### Datasets

For the GB1 DMS dataset of Olson et al. [8], measurements were computed using

36 $${y}_n={\log}_2\frac{\left({c}_n^{\mathrm{out}}+1\right)/\left({c}_{\mathrm{WT}}^{\mathrm{out}}+1\right)}{\left({c}_n^{\mathrm{in}}+1\right)/\left({c}_{\mathrm{WT}}^{\mathrm{in}}+1\right)},$$

where ${c}_n^{\mathrm{in}}$ and ${c}_n^{\mathrm{out}}$ respectively represent the number of reads from the input and output samples (i.e., pre-selection and post-selection libraries), and *n* → WT represents the 55 aa wildtype sequence, corresponding to positions 2–56 of the GB1 domain. To infer the model in Fig. 3a–c and to compute the information metrics in Fig. 3d, only double-mutant sequences with ${c}_n^{\mathrm{in}}\ge 10$ were used; these represent 530,737 out of the 536,085 possible double mutants. For the models in Fig. 3e–g, *y*_*n*_ values for the 1045 single-mutant were also used in the inference procedure.

For the Aβ DMS data of Seuma et al. [9] and TDP-43 DMS data of Bolognesi et al. [10], *y*_*n*_ values respectively represent the nucleation scores and toxicity scores reported by the authors.

For the MPSA of Wong et al. [11], we used the data from replicate 1 of the *BRCA2* minigene library 1. Measurements were computed as

37 $${y}_n={\log}_{10}\left[100\times \frac{\left({c}_n^{\mathrm{inc}}+1\right)/\left({c}_{\mathrm{CONS}}^{\mathrm{inc}}+1\right)}{\left({c}_n^{\mathrm{tot}}+1\right)/\left({c}_{\mathrm{CONS}}^{\mathrm{tot}}+1\right)}\right],$$

where ${c}_n^{\mathrm{inc}}$ and ${c}_n^{\mathrm{tot}}$ respectively represent the number of barcode reads obtained from exon inclusion isoforms and from total mRNA, and *n* → CONS corresponds to the consensus 5′ss sequence CAG/GUAAGU. Corresponding PSI values were computed as ${\mathrm{PSI}}_n={10}^{y_n}$. Only sequences with ${c}_n^{\mathrm{tot}}\ge 10$ were used for inference; these represent 30,483 of the 32,768 possible sequences of the form NNN/GYNNNN.

For the *lac* promoter sort-seq MPRA data of Kinney et al. [12], the {*c*_*my*_} values used for inference represent raw read counts from the “full-wt” experiment. We obtained these data from https://github.com/jbkinney/09_sortseq.

## Supplementary Information

**Additional file 1.** Appendix. Contains a derivation of variational information as a lower bound on predictive information, analyses of multiple simulated data sets, and an in-depth description of the biophysical models featured in Fig. 6.

**Additional file 2.** Peer review history.

## Data Availability

MAVE-NN can be installed from PyPI by executing “pip install mavenn” at the POSIX command line. Comprehensive documentation, including step-by-step tutorials, is provided [50]. Source code, the data sets analyzed in this paper, and the scripts used for training the models and making the figures presented herein, are available under an MIT open-source license [58]. MAVE-NN version 1.0.1 was used for all of the analysis described in this manuscript and is archived on Zenodo [59].
