# Supplementary material for: MAVE-NN: learning genotype-phenotype maps from multiplex assays of variant effect
Source: Genome Biol. 2022 Apr 15;23:98. doi: 10.1186/s13059-022-02661-7 (PMC9011994; doi:10.1186/s13059-022-02661-7)
Supplement: Supplementary file 1 — Additional file 1. Appendix. Contains a derivation of variational information as a lower bound on predictive information, analyses of multiple simulated data sets, and an in-depth description of the biophysical models featured in Fig. 6. [file 13059_2022_2661_MOESM1_ESM.pdf]

Additional file 1: Appendix for "MAVE-NN: learning genotype-phenotype maps from multiplex assays of variant effect"

Ammar Tareen **Mahdi Kooshkbaghi** **Anna Posfai** **William T. Ireland** **David M. McCandlish**

Justin B. Kinney

S1 Variational information as a lower bound on predictive information

Here we show that I_{var} provides a variational lower bound on I_{pre}. This fact was previously described by Barber and Agakov (2003) in the general context of mutual information maximization, then later discussed in the context of MAVE data analysis (Kinney and Atwal, 2014; Atwal and Kinney, 2016). Notably, it also plays an important role in the variational information bottleneck approach to deep learning (Chalk et al., 2016; Alemi et al., 2016). To our knowledge, however, I_{var} itself has not previously been advocated as a useful information-like metric in the context of biological sequence analysis.

As in the main text, let $p_{\text{true}}(y, \phi)$ denote the joint density of y and model-assigned ϕ values that would be observed on test data in the $N \to \infty$ limit, and let $p_{\text{true}}(y|\phi)$ be the corresponding conditional density. Also let $p_{\text{model}}(y|\phi)$ represent the inferred measurement process of the model in question. We then recover **Eq. 31** in Online Methods as follows:

$$
\begin{aligned}
I_{\text{pre}} &= H[y] - H[y|\phi] && \textbf{(S1)} \\
&= H[y] + \langle \log_2 p_{\text{true}}(y|\phi) \rangle_{\text{true}} && \textbf{(S2)} \\
&= H[y] + \langle \log_2 p_{\text{model}}(y|\phi) \rangle_{\text{true}} + \left\langle \log_2 \frac{p_{\text{true}}(y|\phi)}{p_{\text{model}}(y|\phi)} \right\rangle_{\text{true}} \\
&= H[y] - \frac{\log_2(e)}{N} \mathcal{L}_{\text{like}} + D_{\text{KL}}(p_{\text{true}} || p_{\text{model}}) \\
&= I_{\text{var}} + D_{\text{KL}}(p_{\text{true}} || p_{\text{model}}), && \textbf{(S3)}
\end{aligned}
$$

where D_{KL} denotes the Kullback-Leibler divergence, and $\langle \cdot \rangle_{\text{true}}$ indicates averaging over $p_{\text{true}}(y, \phi)$.

Now consider the infinite training data limit. Also assume that the correct G-P map and measurement process are within the class of models considered, as is often the case when analyzing simulated data. Then it is simple to see that minimizing the loss $\mathcal{L}_{\text{like}}$ (or equivalently, maximizing I_{var}) will push $D_{\text{KL}} \to 0$ and $I_{\text{pre}} \to I_{\text{int}}$. For such models, we therefore recover $I_{\text{var}} = I_{\text{pre}} = I_{\text{int}}$.

On finite datasets we must use approximate methods to estimate these information values. The model performance inequality $I_{\text{var}} \leq I_{\text{pre}} \leq I_{\text{true}}$ becomes approximate as a result. Still, the uncertainties in these information quantities are often quite small, due to the large size of typical MAVE datasets, and this model performance inequality can serve well to guide judgements about model accuracy and completeness.

S2 Analysis of simulated GB1 data

Here we assess the performance of MAVE-NN on realistic simulated DMS data, thereby demonstrating its ability to recover ground-truth models when one's modeling assumptions are accurate. First, we trained an additive GE model on the DMS enrichment measurements reported by Olson et al (2014) for 530,737 double mutants of protein GB1. The resulting model was then taken as ground truth and used to simulate new measurements for the same set of variant

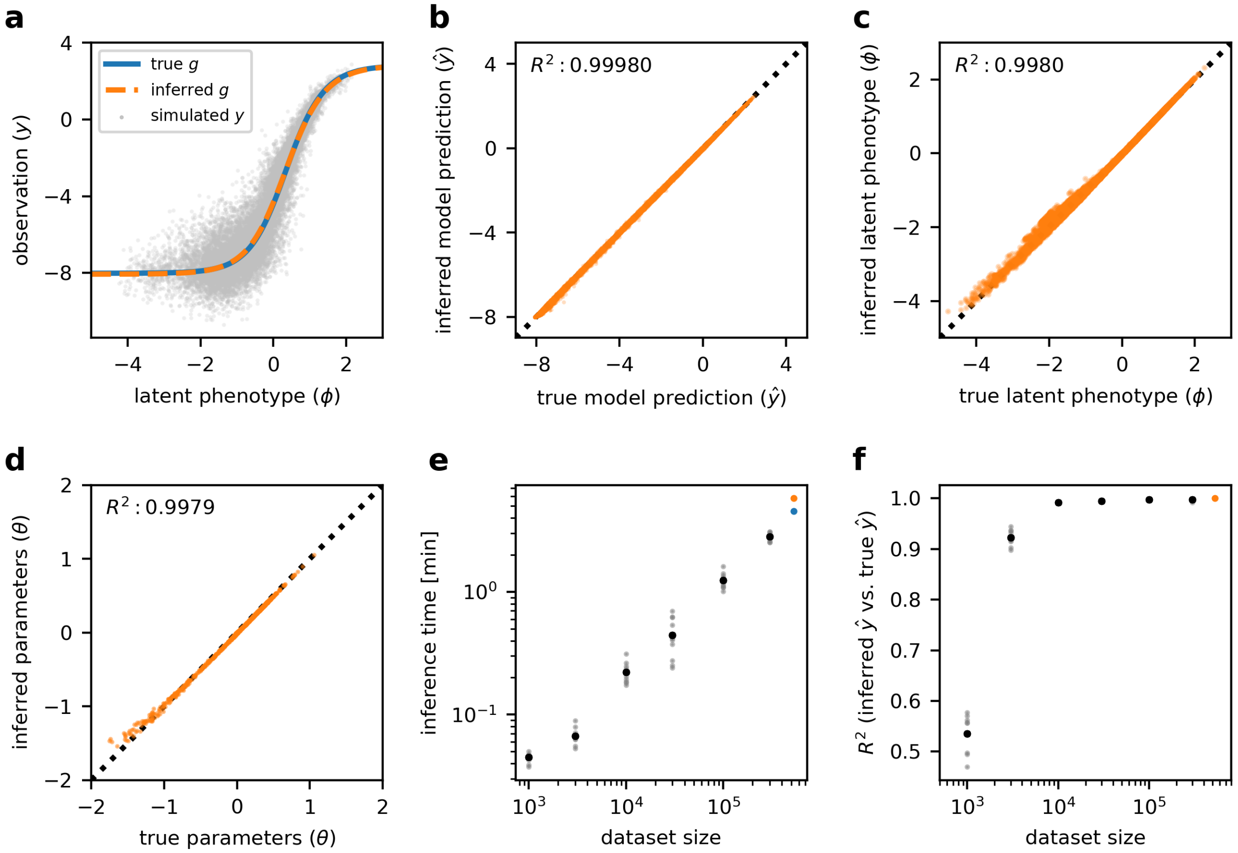

Figure S1: Additive GE models fit to simulated GB1 data. **(a)** GE nonlinearities $g(\cdot)$ for the true model (blue curve) and inferred model (dashed orange curve). Gray dots indicate simulated measurements y plotted against inferred ϕ values. **(b)** Predictions of the inferred vs. true models on held-out simulated test data. **(c)** Latent phenotype predictions of the inferred vs. true model. **(d)** G-P map parameters of the inferred vs. true model. **(e)** Model inference times on simulated datasets of varying size (gray dots). Inference times were recorded for 10 replicate datasets of each size (1×10^3, 3×10^3, 1×10^4, 3×10^4, 1×10^5, or 3×10^5 observations). Black dots indicate the mean inference time for each dataset size. Blue dot indicates the inference time for the original GB1 dataset. Orange dot represents inference time for the model (from panels **a-d**) trained on the full-sized simulated dataset. **(f)** R^2 values comparing the predictions of these inferred models to those of the true model. Black dots indicate mean R^2 values for each dataset size. Orange dot indicates performance of the model trained on the full-sized simulated dataset. Every model inferred for this figure has the same architecture, and all simulated datasets were split the same way as the original dataset into training, validation, and test sets (i.e., 90:5:5). GE: global epistasis; GB1: protein G domain B1; G-P: genotype-phenotype.

sequences. This was done using the model's `simulate_dataset()` method, which is included in all MAVE-NN models to streamline the process of dataset simulation. We then trained a new additive GE model on these simulated data, and compared both the parameters and predictions of this inferred model to those of the true model (**Fig. S1a-d**).

Fig. S1a shows that the inferred GE nonlinearity $g(\cdot)$ agrees remarkably well with the true nonlinearity. Model predictions on held-out test data also correlate highly with the predictions of the true model (**Fig. S1b**). Plotting inferred latent phenotype values against true latent phenotype values reveals a near-perfect correspondence (**Fig. S1c**), and the same is found when comparing inferred G-P map parameters against true G-P map parameters (**Fig. S1d**).

Additionally, we fit additive GE models to simulated datasets of varying size and recorded the model inference time required on one CPU node of a computer cluster. These times are plotted against dataset size in **Fig. S1e**. Model inference for the original published dataset was fast and took only ~ 5 minutes. While the performance of these inferred models improved with increasing simulated dataset size, even moderately sized datasets (e.g., 10,000 observations) yielded models with high correspondence to ground truth (**Fig. S1f**).

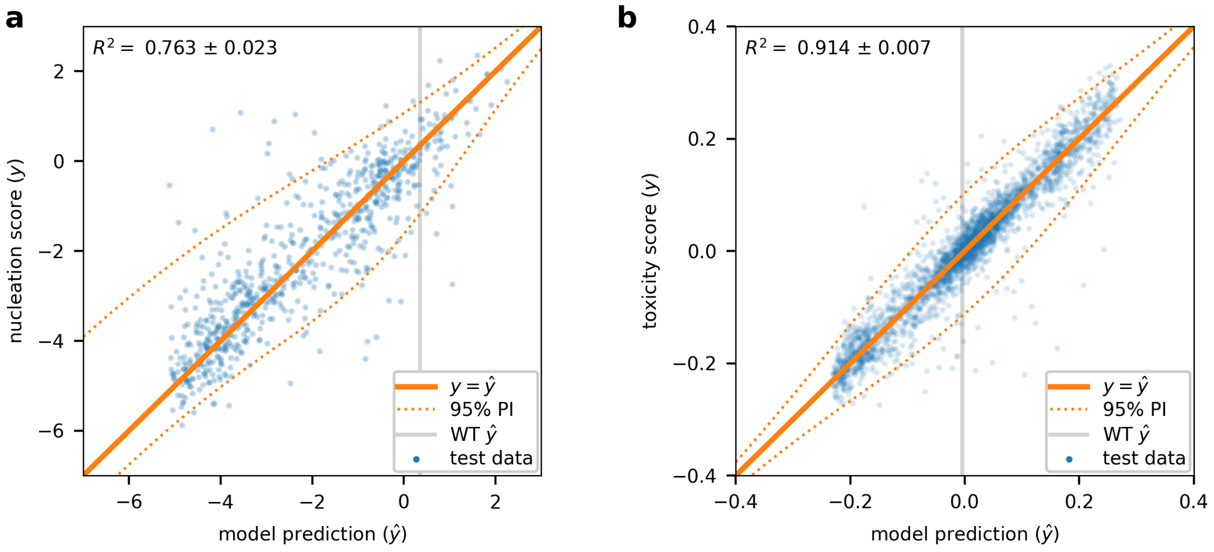

Figure S2: Additional analysis of DMS data for Aβ (Seuma et al., 2021) and for TDP-43 (Bolognesi et al., 2019). **(a)** Nucleation scores vs. predictions for an additive GE model of Aβ. **(b)** Toxicity scores vs. predictions for an additive GE model of TDP-43. DMS: deep mutational scanning; Aβ: amyloid beta; TDP-43: TAR DNA-binding protein 43; GE: global epistasis; PI: prediction interval; WT: wildtype.

S3 Biophysical modeling

Fig. 6 of the main text illustrates two biophysical models trained using MAVE-NN. Here we review the general rationale for models of this form, after which we derive the equations for the two specific models. Please refer to the online documentation for details about how these equations were coded as G-P maps within the MAVE-NN API.

S3.1 General mathematical form of thermodynamic models

Both of the biophysical models featured in **Fig. 6** are examples of thermodynamic models, which are defined by three key assumptions:

1. At each instant in time, the system of interest can occupy one of a fixed number of possible states.
2. The probability of the system being in each state depends on that state's Gibbs free energy via Boltzmann's law, which describes the probability one would observe for a system in thermal equilibrium.
3. The molecular phenotype of interest is a probability-weighted average of the activities of each individual state.

Mathematically, this means that the latent phenotype $\phi(x)$, for a given sequence x, is a weighted sum of state-specific activities:

$$\phi(x) = \sum_s \phi_s(x) p(s|x), \quad \textbf{(S4)}$$

where s indexes possible states of the system, $\phi_s(x)$ is the (generally sequence-dependent) activity of state s, and $p(s|x)$ is the Boltzmann probability of state s. Botlzmann's law tells us that this probability is given by

$$p(s|x) = \frac{1}{Z(x)} \exp\left[-G_s(x)/k_B T\right], \quad \textbf{(S5)}$$

where $G_s(x)$ is the (generally sequence-dependent) Gibbs free energy of state s,

$$Z(x) = \sum_s \exp\left[-G_s(x)/k_B T\right] \quad \textbf{(S6)}$$

is a normalization factor that ensures the probabilities of all states sum to one, $k_B = 1.987 \times 10^{-3} \frac{\text{kcal}}{\text{mol}\cdot\text{K}}$ is Boltzmann's constant, and T is temperature in kelvin. Note that expressing model parameters in units of kcal/mol thus requires

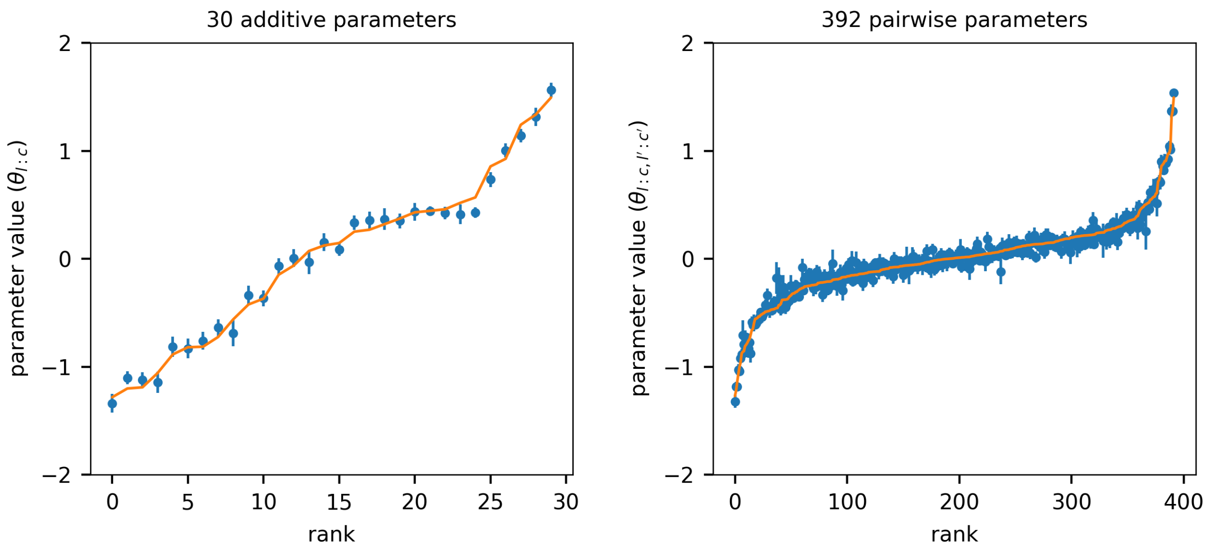

Figure S3: Parameter uncertainties inferred using MAVE-NN's built-in parametric bootstrap functionality. Shown are the mean values (blue dots) and standard deviations (blue lines) of both the additive parameters (left panel) and pairwise parameters (right panel) of the pairwise G-P map inferred from the MPSA data of Wong et al. (2018). In each panel, parameters are arrayed along the horizontal axis according to the rank order of their best-fit values (orange line). The values of these best-fit parameters are illustrated in **Figs. 5e** and **5f** of the main text. G-P: genotype-phenotype; MPSA: massively parallel splicing assay.

knowing the temperature at which the latent phenotype was measured. For example, $k_B T = 0.582\ \frac{\text{kcal}}{\text{mol}}$ at 20 °C (room temperature), whereas $k_B T = 0.616\ \frac{\text{kcal}}{\text{mol}}$ at 37 °C (body temperature).

To infer biophysical models from MAVE data, one must propose specific mathematical formulas for how the state activities $\phi_s(x)$ and Gibbs free energies $G_s(x)$ depend on sequence. These quantities will depend on some set of *a priori* unknown parameters; these are the G-P map parameters θ. The mathematical form of the G-P map itself, $\phi(x;\theta)$, then follows from Eqs. **S4**, **S5**, and **S6**. To use a thermodynamic model as a G-P map within MAVE-NN, users must provide the specific equation for $\phi(x;\theta)$, written in terms of a one-hot encoded sequence vector $\vec{x}$, to the MAVE-NN API. Please refer to the online documentation for details on how to do this.

S3.2 Thermodynamic model for protein GB1

Fig. S4a illustrates the thermodynamic model for protein GB1 that was proposed by Otwinowski (2018) to explain the DMS data of Olson et al. (2014). This model assumes three possible states for GB1: (1) unfolded, (2) folded but not bound to IgG, and (3) folded and bound to IgG. The activity in question, $\phi(x)$, is the fraction of time a GB1 molecule with peptide sequence x is bound to IgG. The corresponding state-specific activities are $\phi_{(1)} = 0$, $\phi_{(2)} = 0$, and $\phi_{(3)} = 1$. Note that, both here and in the models below, we assume that all ϕ_s are fixed binary numbers that are known *a priori*, i.e., they do not depend on sequence x or on any trainable parameters in θ.

The unfolded state (1) is taken to be the reference state and assigned a Gibbs free energy of 0. ΔG_{F} denotes the Gibbs free energy of the folded state relative to the unfolded state, and thus state (2) has energy ΔG_{F}. ΔG_{B} is the Gibbs free energy change upon the binding of a folded GB1 molecule to IgG, and so the energy of state (3) is $\Delta G_{\text{F}} + \Delta G_{\text{B}}$. Each of these energies is assumed to be an additive function of the GB1 sequence x; the specific mathematical formulas for ΔG_{F} and ΔG_{B} in terms of the one-hot encoded vector $\vec{x}$ are as shown. The parameters that control these two energies are $\vec{\theta}_{\text{F}}$, θ^0_{F} (for ΔG_{F}) and $\vec{\theta}_{\text{B}}$, θ^0_{B} (for ΔG_{B}). The resulting set of trainable G-P map parameters is

$$\theta = \left\{ \theta^0_{\text{F}}, \vec{\theta}_{\text{F}}, \theta^0_{\text{B}}, \vec{\theta}_{\text{B}} \right\}. \quad \textbf{(S7)}$$

To infer values for these parameters using MAVE-NN, we paired the biophysical G-P map $\phi(x;\theta)$ with a GE measurement process $p(y|\phi)$ having a trainable nonlinearity and a heteroscedastic skewed-t noise model. The resulting latent phenotype model was trained on the DMS data of Olson et al. (2014).

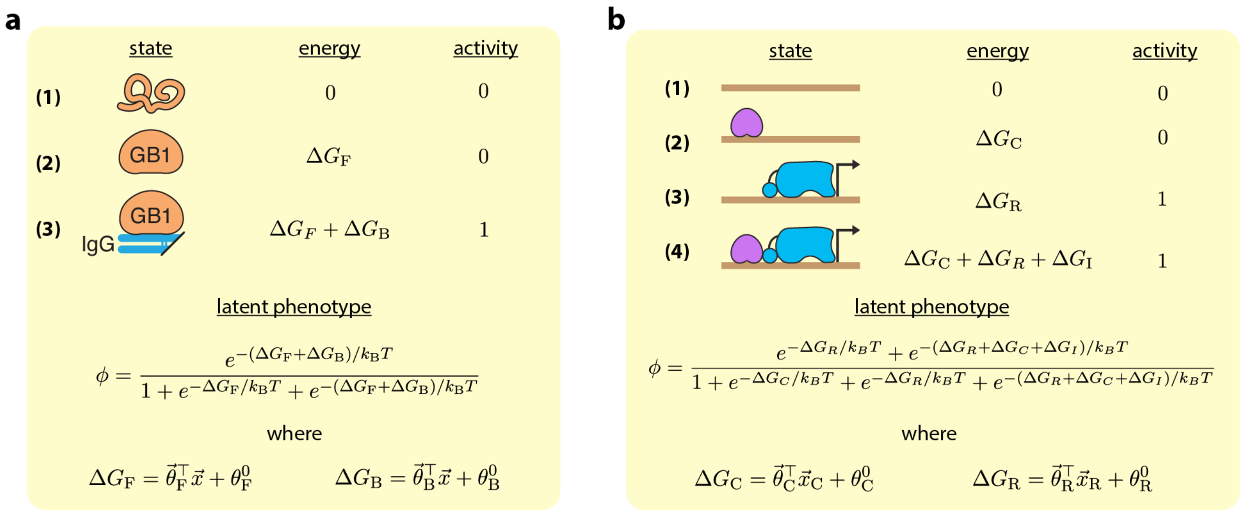

Figure S4: Thermodynamic models featured in **Fig. 6** of the main text. **(a)** Three-state model for GB1 folding and binding to IgG. $\vec{x}$ denotes the one-hot encoding of a variant GB1 protein sequence. The trainable G-P map parameters are $\vec{\theta}_{\rm F}$, $\theta^0_{\rm F}$, $\vec{\theta}_{\rm B}$, and $\theta^0_{\rm B}$. **(b)** Four-state model for transcriptional regulation of the *E. coli lac* promoter by CRP (purple) and RNAP (blue). Variable promoter DNA is indicated in brown. $\vec{x}_C$ and $\vec{x}_R$ respectively denote one-hot encodings of a variant 26 nt CRP binding site and a variant 41 nt RNAP binding site. The trainable G-P map parameters are $\vec{\theta}_{\rm C}$, $\theta^0_{\rm C}$, $\vec{\theta}_{\rm R}$, $\theta^0_{\rm R}$, and $\Delta G_{\rm I}$. GB1: protein G domain B1; IgG: immunoglobulin G; G-P: genotype-phenotype; CRP: cAMP receptor protein; RNAP: σ^{70} RNA polymerase.

The resulting values for $\vec{\theta}_{\rm F}$ and $\vec{\theta}_{\rm B}$ are illustrated as heatmaps in **Fig. 6b** of the main text. These are expressed in the "wildtype" gauge, i.e., θ^0_F and θ^0_B are the folding and binding energies of the wildtype GB1 sequence, while the elements of the vectors $\vec{\theta}_{\rm F}$ and $\vec{\theta}_{\rm B}$ describe the energetic effects of single amino acid mutations. All of these parameter values represent Gibbs free energies computed using $k_BT = 0.582\,\frac{\rm kcal}{\rm mol}$, since the RNA display experiment of Olson et al. (2014) was performed at room temperature.

S3.3 Thermodynamic model for the *lac* promoter

Fig. S4b illustrates a thermodynamic model for transcriptional activation at the *lac* promoter of *Escherichia coli*. This model describes the DNA binding energies of two transcription factors, the cAMP receptor protein (CRP) and σ^{70} RNA polymerase (RNAP), as well as a cooperative interaction between these two proteins that occurs when both are bound to promoter DNA. This model, which was originally proposed by Kinney et al. (2010), was trained on data from their sort-seq MPRA, in which a 75 bp region spanning the CRP and RNAP binding sites of the wildtype *lac* promoter was mutagenized at 12% per nucleotide, resulting in $\sim$9 mutations per sequence on average.

In this model, promoter DNA is assumed to be in one of four possible states: (1) empty, (2) bound by CRP, (3) bound by RNAP, and (4) bound by both CRP and RNAP. State (1) is taken to be the reference state. $\Delta G_{\rm C}$ denotes the Gibbs free energy of CRP binding to DNA, and $\Delta G_{\rm R}$ is the energy of RNAP binding to DNA. Both of these quantities are assumed to be additive functions of the lac promoter sequence x. More specifically, $\Delta G_{\rm C}$ is assumed to depend only on a 26 nt subsequence (the CRP binding site), the one-hot encoding of which we denote by $\vec{x}_{\rm C}$. Similarly, $\Delta G_{\rm R}$ is taken to depend on a 41 nt subsequence $\vec{x}_R$ representing the RNAP binding site. The corresponding parameters of these additive models are $\theta^0_{\rm C}$, $\vec{\theta}_{\rm C}$ for CRP, and $\theta^0_{\rm R}$, $\vec{\theta}_{\rm R}$ for RNAP. $\Delta G_{\rm I}$ is the Gibbs free energy of interaction between DNA-bound CRP and DNA-bound RNAP, and is assumed to be independent of sequence. The latent phenotype of interest, ϕ, is the fraction of time that RNAP is bound to promoter DNA. We assume this quantity is proportional to the rate of transcript initiation and thus the level of gene expression. The state-specific activities are therefore fixed and given by $\phi_{(1)} = \phi_{(2)} = 0$ and $\phi_{(3)} = \phi_{(4)} = 1$. The resulting set of trainable G-P map parameters is

$$\theta = \left\{\theta^0_{\rm C}, \vec{\theta}_{\rm C}, \theta^0_{\rm R}, \vec{\theta}_{\rm R}, \Delta G_{\rm I}\right\}. \tag{S8}$$

This biophysical G-P map, $\phi(x;\theta)$, was paired with an MPA measurement process and trained on the data of Kinney et al. 2010. The resulting inferred values for $\vec{\theta}_{\rm C}$ and $\vec{\theta}_{\rm R}$ are illustrated in **Fig. 6d** as sequence logos; the inferred value of

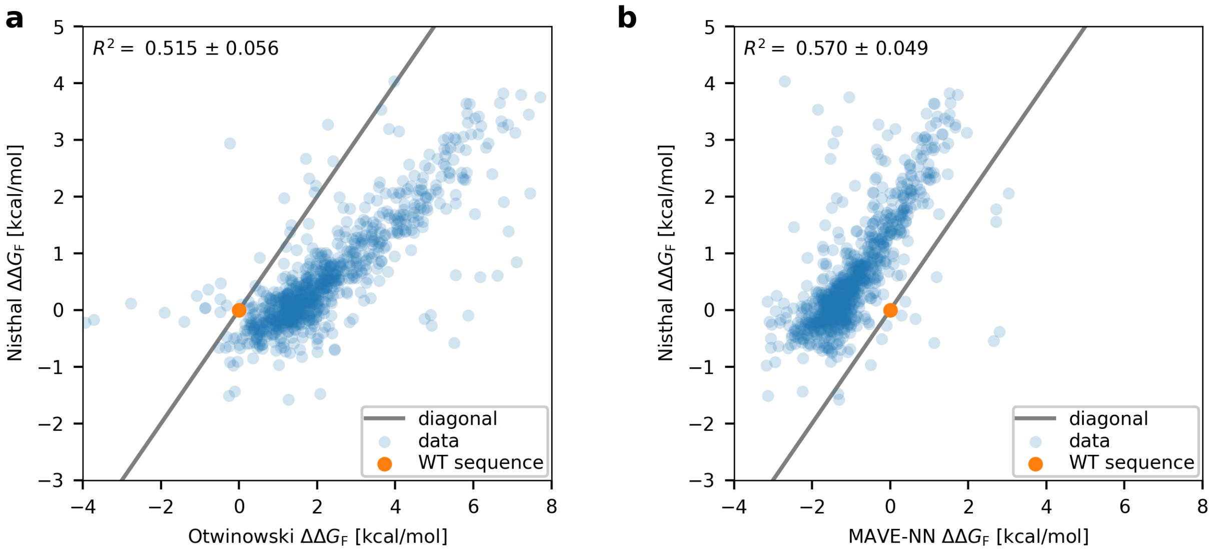

Figure S5: Gibbs free energies of folding ($\Delta\Delta G_{\mathrm{F}}$) measured by Nisthal et al. (2019), compared to predictions made by the thermodynamic models inferred by **(a)** Otwinowski (2018) and **(b)** MAVE-NN. WT: wildtype.

ΔG_{I} is also shown. The standard errors on ΔG_{I} were estimated by repeating the inference procedure on 10 simulated datasets using the inferred model's built-in `bootstrap()` method.
