# Supplementary material for: MAVE-NN: learning genotype-phenotype maps from multiplex assays of variant effect
Source: Genome Biol. 2022 Apr 15;23:98. doi: 10.1186/s13059-022-02661-7 (PMC9011994; doi:10.1186/s13059-022-02661-7)
Supplement: Supplementary file 2 — Additional file 2. Peer review history. [file 13059_2022_2661_MOESM2_ESM.docx]

**Review history**

**First round of review**

**Reviewer 1**

1) General comments

This manuscript described MAVE-NN, a new method for modelling genotype-phenotype relationships from multiplex assay of variant effect (MAVE) data in both coding and noncoding contexts. In addition to modelling the underlying phenotype driving the functional scores in an MPRA or DMS experiment, the authors show that MAVE-NN can be used to transform functional scores into more meaningful biophysical properties, such as Gibbs free energy. This has the potential to address a major weakness of MAVE methods in general, namely that MAVE functional scores are essentially unitless abstractions quantifying "enrichment" or "activity" in a complex experimental context.

However, despite the authors claims that the software is easy to use, it is not clearly explained how one could go about applying this to other datasets. I think the authors could either re-frame the paper as a theoretical advance, that would be much less impactful for other people in the field, or provide a substantial amount of additional documentation to make this a software tool that others can use on their own data.

2) Specific comments for revision

a) Major comments

i) I had no problem installing the software and running the example data, but it was not clear from the documentation how to format a new dataset. The authors have put an admirable amount of effort into packaged tutorials and example datasets, but there's no part of the documentation (or manuscript) that steps through how someone with a moderate level of scientific Python experience and a MAVE dataset in hand would go about formatting the input files, choosing a model, and running MAVE-NN. I'm sure that one could adapt the tutorials with some effort, but the input file requirements and general advice for choosing an appropriate G-P map type should be clearly stated in generic terms. Without this, I think the utility of the software will be rather limited.

ii) In the abstract and through the introduction, the authors imply that MAVE-NN is a generic and widely-applicable method for MPRA and DMS experiments. This seems to be true, but there are two important caveats that should be disclosed much earlier, probably at the end of the introduction where the method is introduced. These are that MAVE-NN requires the presence of some double-mutants in the variant library and that MAVE-NN requires all input sequences to have the same length.

The fact that double-mutants are required doesn't come up until the bottom of page 8, and is a very important limitation of the method, given that experiments designed with clinical applications in mind specifically exclude double-mutants, and some only include single-nucleotide mutants. The observation that MAVE-NN's good performance with a very small number of double mutants has major implications for future DMS experimental designs is a good one and could be expanded upon.

The requirement that all sequences have the same length is stated in the G-P maps section of the online methods, and is important because some DMS techniques (notably the popular MITE-seq method) use shotgun sequencing and therefore do not resolve full-length variants. While it is possible to generate full-length variant sequences from shotgun data by assuming all unobserved bases are wild-type, this is not a good assumption. Perhaps this is something that MAVE-NN could model explicitly in a future version if the authors are interested.

iii) I struggled to understand how the biophysical model for GB1 was specified and constructed. There is a little bit of extra information about the specific models shown in the paper in Figure S4, but the methods section only says that:
"MAVE-NN further supports custom G-P maps that users can define by subclassing the G-P map base class. These G-P maps can have arbitrary functional form, e.g., representing specific biophysical hypotheses of sequence function."

I went looking through the source code and found https://github.com/jbkinney/mavenn/blob/master/mavenn/examples/other/custom_gpmap/custom_gpmap_three_state_thermodynamic_model_GB1.ipynb but the code comments here are pretty minimal, and the plots in that notebook didn't seem to match what is in Figure 6b, so I wasn't sure I was on the right track.

There is really not enough information at present for someone with MAVE data in hand to be able to use this part of the software, and as far as I could tell, it's not addressed at all in the MAVE-NN documentation. This feature is what makes MAVE-NN so compelling to me and transforms it from what is essentially a normalization method to something much greater. If MAVE-NN is going to be presented as a software package, the authors should do whatever they can to make this capability more accessible to potential users by providing documented examples, generalizable instructions, and other tips to get people going.

b) Minor comments

i) In the Aβ and TDP-43 data, are there any missing values in these datasets and in Figures 4a and 4c? There are quite a few pure white patches that suggest data might be missing, but in the color scheme white indicates no effect. I note that there aren't enough single mutants in these datasets to fill out all squares of their respective heatmaps. The number of missing data values should be reported and they should be clearly identifiable in the figure, maybe by coloring the boxes grey or some other shade that's not in the color map being used.

ii) It's not clear in the methods how the data in https://github.com/jbkniney/09_sortseq was converted into the format required for MAVE-NN, although I have no doubt the required information is in there somewhere.

iii) The authors should consider changing the color map for Figure 6b since the heatmap is showing ΔΔG values instead of Δϕ.

**Reviewer 2**

The authors describe MAVE-NN, a Python package that implements an approach for fitting 'global epistasis' functions from mutational scanning/MPRA experiments.
In general we found this an interesting approach and the tool is likely to be useful for many groups woking with MAVE data - both to fit or remove experimental and biological non-linearities and to fit simple mechanistic (thermodynamic) models. The manuscript is clearly written, although in places it could be improved for an experimental genomics rather than statistical audience - additional explanation can only help in this respect.


Queries and suggestions

Does MAVENN take into account phenotype / measurement error?

Does MAVENN provide confidence intervals for model parameters?

Can MAVENN be used when measurements for two or more different phenotypes are made for each genotype (for example, activity and stability of a protein, or baseline and induced expression for a promoter)?

Free energies in figure S5 and figure 6b do not correspond - if they used the convention of Nisthal et al. in supplementary figure S5 the authors should clarify this

What is the justification for the different training, validation and test splits i.e. 60:20:20, 90:5:5, 80:10:10?

Line 223 and Fig. S2 - how does MAVENN's ability to ''recover ground-truth parameters from simulated datasets" implies that pairwise epistatic terms have been "precisely determined" for the massively parallel splicing assay?

The authors should consider including a cell growth rate/ competition based assay in Figure 1

The last sentence of the abstract ("MAVE-NN thus provides a unified solution to a major outstanding need in the MAVE community.") is rather grandiose / self-aggrandising and could be edited for modesty.

Intro line 81 "Neighbor G-P maps incorporate interactions between nearest-neighbor characters" - I think neighbor and neighbor characters will need defining for a general audience here.

Many readers of this journal will not be familiar with the intrinsic information as a metric, so the values of Ivar and Ipre will not mean much without an explanation of what a value of e.g. 2.194 actually means.

Line 184 - should probably clarify what the selections were for (amyloid beta - amyloid nucleation, tdp43 - toxicity; they were not the same). Minor: TDP-43 wasn't mutated using mutagenic pcr but by error-prone 'doped' oligonucleotide synthesis.

Typos:

* Line 119 "equality obtains" => "obtains equality"
* Line 140 "Inspired in by" => "Inspired by"
* Line 145 "critical GB1 residues" are these binding site / ligand proximal positions?
* Line 161 "0.008 bits and" => "0.008 bits"
* Line 188 "mutagenic PCR" was used for the amyloid beta library; doped oligo synthesis was use for the TDP-43 library
* Line 239 "intended use by" => "intended for use by"
* Line 254 "predictions our model" => "predictions of our model"

**Authors’ response to reviewers**

# Reviewer #1 Critique:

1) General comments:

This manuscript described MAVE-NN, a new method for modelling genotype-phenotype relationships from multiplex assay of variant effect (MAVE) data in both coding and noncoding contexts. In addition to modelling the underlying phenotype driving the functional scores in an MPRA or DMS experiment, the authors show that MAVE-NN can be used to transform functional scores into more meaningful biophysical properties, such as Gibbs free energy. This has the potential to address a major weakness of MAVE methods in general, namely that MAVE functional scores are essentially unitless abstractions quantifying "enrichment" or "activity" in a complex experimental context.

We thank the referee for this favorable assessment.

However, despite the authors claims that the software is easy to use, it is not clearly explained how one could go about applying this to other datasets. I think the authors could either re-frame the paper as a theoretical advance, that would be much less impactful for other people in the field, or provide a substantial amount of additional documentation to make this a software tool that others can use on their own data.

This feedback was very helpful. We fully intend for MAVE-NN to be a practical software package that other research groups find useful. We have therefore made substantial changes to both the manuscript and the online documentation. Please see our detailed responses below.

2) Specific comments for revision
   a) Major comments
i) I had no problem installing the software and running the example data, but it was not clear from the documentation how to format a new dataset. The authors have put an admirable amount of effort into packaged tutorials and example datasets, but there's no part of the documentation (or manuscript) that steps through how someone with a moderate level of scientific Python experience and a MAVE dataset in hand would go about formatting the input files, choosing a model, and running MAVE-NN. I'm sure that one could adapt the tutorials with some effort, but the input file requirements and general advice for choosing an appropriate G-P map type should be clearly stated in generic terms. Without this, I think the utility of the software will be rather limited.

We have substantially revised the online documentation at https://mavenn.readthedocs.io/ to address this critique. In particular:

1. The “Built-in datasets” section of the documentation steps users through the reformatting of previously published datasets for analysis using MAVE-NN.
2. The “Modeling tutorials” section of the documentation steps users through the process of defining a model, the rationales used for choosing model architecture, the model training process, and the assessment / visualization of the resulting models.

In this documentation, we have tried to provide tips for formatting data and choosing model architecture where appropriate. In terms of the reviewer’s specific point about providing guidance for users on a choice of model, our view is that best practices for modeling MAVE data have not yet emerged, and that in particular there isn’t community consensus on which mathematical forms one should use to model G-P maps in different situations using different datasets. Indeed, one of the primary purposes of MAVE-NN is to provide users with tools that will help them explore a wide range of G-P map modeling strategies. Thus, we advise users to explore multiple modeling strategies during the initial phases of data analysis, and to aid in this we provide modeling tutorials that walk users step-by-step through a diverse set of modeling applications.

ii) In the abstract and through the introduction, the authors imply that MAVE-NN is a generic and widely-applicable method for MPRA and DMS experiments. This seems to be true, but there are two important caveats that should be disclosed much earlier, probably at the end of the introduction where the method is introduced. These are that MAVE-NN requires the presence of some double-mutants in the variant library and that MAVE-NN requires all input sequences to have the same length.

The fact that double-mutants are required doesn't come up until the bottom of page 8, and is a very important limitation of the method, given that experiments designed with clinical applications in mind specifically exclude double-mutants, and some only include single-nucleotide mutants.

The issue of how to handle libraries that only contain single-mutation variants is an interesting one, and was not discussed sufficiently in the original manuscript. We address this issue more directly in the revised manuscript (lines 106-113, 329-346). We have also revised MAVE-NN so that it can indeed handle libraries of only single-mutation variants, and we have updated the online documentation accordingly. We emphasize, however, that the analysis of single-mutation libraries places important restrictions on the types of models that one can meaningfully train, and that these restrictions are not a shortcoming of the MAVE-NN software package per se, but rather reflect fundamental mathematical issues of parameter non-identifiability.

The observation that MAVE-NN's good performance with a very small number of double mutants has major implications for future DMS experimental designs is a good one and could be expanded upon.

We agree that this important point could be expanded on. The revised manuscript does so (lines 62-69; 338- 346), and draws a direct connection between the benefits of having a small number of double mutants and the restrictions that must be placed on models trained on libraries of only single-mutation variants.

The requirement that all sequences have the same length is stated in the G-P maps section of the online methods, and is important because some DMS techniques (notably the popular MITE-seq method) use shotgun sequencing and therefore do not resolve full-length variants. While it is possible to generate full-length variant sequences from shotgun data by assuming all unobserved bases are wild-type, this is not a good assumption. Perhaps this is something that MAVE-NN could model explicitly in a future version if the authors are interested.

We do plan to expand the capabilities of MAVE-NN in the near future, and this is a good suggestion for one such enhancement. The primary issue here, as we see it, is how to perform quantitative model inference when the assayed sequences are only partially known. However, this is a nontrivial mathematical problem in its own right, and in particular it falls outside our formalization of the G-P map inference problem via latent phenotype models. As such, we believe this question deserves substantially more attention than we would be able to devote to it in the present context. We thus view adding this capability as being beyond the scope of the current manuscript.

There is the second issue of whether MAVE-NN can handle perfectly known sequences of varying length. We have decided not to support this in the initial version of MAVE-NN for two reasons: (a) Sequences are almost always assayed within larger sequence contexts, the sequences of which can be used to pad core sequences, thus making them the same length and compatible with MAVE-NN. (b) None of the built-in model types that are currently implemented are compatible with variable length sequence libraries.

iii) I struggled to understand how the biophysical model for GB1 was specified and constructed. There is a little bit of extra information about the specific models shown in the paper in Figure S4, but the methods section only says that:

"MAVE-NN further supports custom G-P maps that users can define by subclassing the G-P map base class. These G-P maps can have arbitrary functional form, e.g., representing specific biophysical hypotheses of sequence function."

I went looking through the source code and found at [URL] but the code comments here are pretty minimal, and the plots in that notebook didn't seem to match what is in Figure 6b, so I wasn't sure I was on the right track.

There is really not enough information at present for someone with MAVE data in hand to be able to use this part of the software, and as far as I could tell, it's not addressed at all in the MAVE-NN documentation. This feature is what makes MAVE-NN so compelling to me and transforms it from what is essentially a normalization method to something much greater. If MAVE-NN is going to be presented as a software package, the authors should do whatever they can to make this capability more accessible to potential users by providing documented examples, generalizable instructions, and other tips to get people going.

We thank the referee for underscoring the importance of MAVE-NN’s biophysical modeling capabilities. We have added a section to Supplemental Information, titled “Biophysical modeling using MAVE-NN”, that describes how biophysical models like those in Fig. 6 are constructed and implemented. We have also added a tutorial, “Tutorial 4: Protein DMS modeling using a biophysical G-P map”, to the “Modeling Tutorials” section of the online documentation. This tutorial steps users through the process of defining a biophysical model through subclassing, as well as analyzing the parameters and predictions of the trained model. That being said, biophysical G-P maps are inherently more complex than additive or pairwise G-P maps, as the mathematical forms of these models will vary from application to application. For this reason, we have yet to identify a way of supporting this capability with built-in G-P maps as opposed to custom G-P maps defined by the user.

b) Minor comments

i) In the Aβ and TDP-43 data, are there any missing values in these datasets and in Figures 4a and 4c? There are quite a few pure white patches that suggest data might be missing, but in the color scheme white indicates no effect. I note that there aren't enough single mutants in these datasets to fill out all squares of their respective heatmaps. The number of missing data values should be reported and they should be clearly identifiable in the figure, maybe by coloring the boxes grey or some other shade that's not in the color map being used.

We have adjusted the color maps in Figs. 3 and 4 to better contrast these missing values with parameter values of zero. We have also updated the Fig. 4 legend to list the number of missing parameter values, and to state explicitly that missing values are shown in white.

ii) It's not clear in the methods how the data in https://github.com/jbkinney/09_sortseq was converted into the format required for MAVE-NN, although I have no doubt the required information is in there somewhere.

We have expanded our explanation in the MAVE-NN online documentation of how we processed raw data from https://github.com/jbkinney/09_sortseq for input into MAVE-NN. This documentation (https://mavenn.readthedocs.io/en/latest/datasets/dataset_sortseq.html) comes in the form of a Jupyter notebook with explicit code for doing this reformatting. Similar documentation and code has been provided for other datasets in this publication as well (https://mavenn.readthedocs.io/en/latest/datasets.html).

iii) The authors should consider changing the color map for Figure 6b since the heatmap is showing ΔΔG values instead of Δϕ.

This is a good suggestion. The colormaps in Fig. 6b have been changed accordingly.

# Reviewer #2 Critique:

The authors describe MAVE-NN, a Python package that implements an approach for fitting 'global epistasis' functions from mutational scanning/MPRA experiments. In general we found this an interesting approach and the tool is likely to be useful for many groups working with MAVE data - both to fit or remove experimental and biological non-linearities and to fit simple mechanistic (thermodynamic) models. The manuscript is clearly written, although in places it could be improved for an experimental genomics rather than statistical audience - additional explanation can only help in this respect.

We thank the reviewer for this positive assessment. We believe our revisions to both the manuscript and online documentation will make the manuscript more accessible to investigators working in experimental genomics, who indeed make up much of our intended audience.

Queries and suggestions

Does MAVENN take into account phenotype / measurement error?

MAVE-NN, as initially reported, does indeed account for measurement error, in that the quantitative form of measurement error is inferred from the measurements themselves via the “measurement process” component of the latent phenotype model. Here, however, the reviewer appears to be asking specifically whether MAVE- NN can handle user-supplied uncertainties on experimental measurements. Our original version of MAVE-NN did not support this, but the revised MAVE-NN package does. Specifically, we have added support for an “Empirical” noise model within the context of a GE measurement process. The “Empirical” noise model assumes that measurement uncertainties have a Gaussian form with fixed, user-supplied standard errors. This is described in lines 615-625 of the revised text.

Does MAVENN provide confidence intervals for model parameters?

We have augmented MAVE-NN to estimate model uncertainties using a parametric bootstrap approach. Specifically, after the best-fit model has been identified, users now have the option to call a function that samples other plausible models by first simulating datasets using the best-fit model as ground truth, then re- inferring models from these simulated datasets. This is described in lines 744-767 of the revised text. We illustrate this capability in the revised Fig. S3, which shows confidence intervals thus determined for the additive and pairwise parameters of a pairwise GE regression model fit to the splicing MPRA data of Wong et al. (2018). We also use this approach to compute uncertainties in the protein-protein interaction energy of a biophysical model fit to the *lac* promoter sort-seq MPRA dataset (Figs. 6e)

We note, however, that there is an important caveat to this method of computing parameter uncertainties. G-P maps typically exhibit many gauge (i.e., non-identifiable) degrees of freedom that have to be “fixed” before comparing the values of parameters from two or more models. For models with linear G-P maps (as in the splicing MPRA analysis), the built-in gauge-fixing procedure described in the section “Gauge modes and diffeomorphic modes” (lines 530-575) of the main text provides one way to deal with this issue. But for other types of G-P maps, such as custom G-P maps representing biophysical models, we have yet to find a one- size-fits-all solution to this gauge-fixing problem, and there is likely to be no reasonable procedure for estimating parameter uncertainty for overparameterized neural network models (including the “blackbox” models that MAVE-NN supports). We therefore leave it to the user to decide how best to convert this set of simulation-inferred models that MAVE-NN provides into explicit confidence intervals for G-P map parameters.

Can MAVENN be used when measurements for two or more different phenotypes are made for each genotype (for example, activity and stability of a protein, or baseline and induced expression for a promoter)?

This is a very good question. The ability to do this type of multi-target regression is indeed important for the analysis of MAVE data. In its current form, MAVE-NN does not have this capability because we first wanted to make sure that we provided robust and user-friendly support for single-target regression, and enabling multi- target regression would substantially complicate the user interface. However, multi-target regression is fully compatible with the form of latent phenotype models on which MAVE-NN is based, and we intend for an updated version of MAVE-NN to support this type of inference in the near future.

Free energies in figure S5 and figure 6b do not correspond - if they used the convention of Nisthal et al. in supplementary figure S5 the authors should clarify this

We thank the referee for pointing out this oversight. We have adopted the more standard convention (not that of Nisthal et al.) that increasing stability corresponds to decreasing folding energy, with the WT sequence having a folding energy of ΔΔ𝐺_!_ = 0. Fig. S5 has been updated to reflect this.

What is the justification for the different training, validation and test splits i.e. 60:20:20, 90:5:5, 80:10:10?

These choices of split were mostly arbitrary. We did not systematically tune these hyperparameters, nor does the specific choice of split appear to make a big difference in any of our key results.

Line 223 and Fig. S2 - how does MAVENN's ability to ''recover ground-truth parameters from simulated datasets" implies that pairwise epistatic terms have been "precisely determined" for the massively parallel splicing assay?

This reflects MAVE-NN’s parametric bootstrap approach to quantifying the precision of G-P map parameter estimates. Note that we are not claiming that the pairwise epistatic terms are “accurate”, only that MAVE-NN can precisely recover parameter values that are near to “ground truth values” under the assumption that the functional form of the latent phenotype model is correct. To help avoid any misunderstanding, we have clarified this in lines 744-767 of the revised text.

The authors should consider including a cell growth rate/ competition based assay in Figure 1.

This is a good suggestion. We have revised Fig. 1a to illustrate the selective growth DMS approach used in Seuma et al. (2021) and Bolognesi et al. (2019), the analyses of which are shown in Fig. 4.

The last sentence of the abstract ("MAVE-NN thus provides a unified solution to a major outstanding need in the MAVE community.") is rather grandiose / self-aggrandizing and could be edited for modesty.

It was not our intention to claim that MAVE-NN provides a complete solution to the MAVE community’s quantitative modeling problems, only that it provides a conceptually coherent way of addressing some important needs. This sentence has been changed to be more specific.

Intro line 81 "Neighbor G-P maps incorporate interactions between nearest-neighbor characters" - I think neighbor and neighbor characters will need defining for a general audience here.

The explanation of neighbor G-P maps has been clarified (lines 83-86).

Many readers of this journal will not be familiar with the intrinsic information as a metric, so the values of Ivar and Ipre will not mean much without an explanation of what a value of e.g. 2.194 actually means.

The revised text more expansively motivates mutual information and provides an expanded explanation for how to interpret mutual information values (lines 118-133).

Line 184 - should probably clarify what the selections were for (amyloid beta - amyloid nucleation, tdp43 - toxicity; they were not the same).

This is a good suggestion, especially because the selections in these experiments are not obvious. We have expanded this description (lines 207-210).

Minor: TDP-43 wasn't mutated using mutagenic pcr but by error-prone 'doped' oligonucleotide synthesis.

We apologize for this error; it has been corrected in the revised manuscript (lines 214-215).

Typos:

- Line 119 "equality obtains" =>
- "obtains equality"
- We have reworded this (line 142).
- Line 140 "Inspired in by" => Inspired by"

Fixed (line 163).

- Line 145 "critical GB1 residues" are these binding site / ligand proximal positions?

We have added text clarifying the structural context of these residues (lines 167-169).

- Line 161 "0.008 bits and" => "0.008 bits"

Fixed.

- Line 188 "mutagenic PCR" was used for the amyloid beta library; doped oligo synthesis was use for the TDP-43 library

Fixed (line 214-215).

- Line 239 "intended use by" => "intended for use by"

Fixed (line 267).

- Line 254 "predictions our model" => "predictions of our model" Fixed (lines 284-285).

**Second round of review**

**Reviewer 1**

I thank the authors for their thoughtful response to my review. They addressed all of my feedback in full and I appreciate the effort this must have taken, particularly expanding the software documentation. I have no concerns regarding the Belliveau dataset that was removed in the revision.

This is a very nice paper describing a potentially hugely impactful method, and I hope that researchers generating DMS data take the suggestion to include a few double mutants so that this and related future methods can be brought to bear. I look forward to spending more time with this software in the future and applying it to some additional datasets.

**Reviewer 2**

The authors have done a great job at addressing and implementing all suggestions.

One minor query concerns the approach taken to estimate confidence intervals for model parameters. Rather than refitting the model using bootstraps of the real training data they instead use the best fitted model as "ground truth" to then generate simulated data matching the training sequences. It's not clear to us why they took this approach and it would be helpful if the authors could briefly explain this / how it would be different to the more obvious (to us) alternative of subsampling the "real" training data.

**Authors’ response to reviewers**

"Reviewer #1: I thank the authors for their thoughtful response to my review. They addressed all of my feedback in full and I appreciate the effort this must have taken, particularly expanding the software documentation. I have no concerns regarding the Belliveau dataset that was removed in the revision. This is a very nice paper describing a potentially hugely impactful method, and I hope that researchers generating DMS data take the suggestion to include a few double mutants so that this and related future methods can be brought to bear. I look forward to spending more time with this software in the future and applying it to some additional datasets.”

We thank the reviewer for this kind assessment.

"Reviewer #2: The authors have done a great job at addressing and implementing all suggestions. One minor query concerns the approach taken to estimate confidence intervals for model parameters.  Rather than refitting the model using bootstraps of the real training data they instead use the best fitted model as "ground truth" to then generate simulated data matching the training sequences.  It's not clear to us why they took this approach and it would be helpful if the authors could briefly explain this / how it would be different to the more obvious (to us) alternative of subsampling the "real" training data.”

We have added an expanded description, in Online Methods, for why we use this parametric bootstrap approach instead of the more standard pair-sampling bootstrap approach referred to by the referee.
